# Genomic and Physiological Characterization of *Metabacillus flavus* sp. nov., a Novel Carotenoid-Producing *Bacilli* Isolated from Korean Marine Mud

**DOI:** 10.3390/microorganisms10050979

**Published:** 2022-05-07

**Authors:** Chi Young Hwang, Eui-Sang Cho, Deok Jun Yoon, In-Tae Cha, Dong-Hyun Jung, Young-Do Nam, So-Lim Park, Seong-Il Lim, Myung-Ji Seo

**Affiliations:** 1 Department of Bioengineering and Nano-Bioengineering, Incheon National University, Incheon 22012, Korea; hcyoung28@gmail.com (C.Y.H.); whdmltkd123@gmail.com (E.-S.C.); deockjoon.yoon@gmail.com (D.J.Y.); 2 Microorganism Resources Division, National Institute of Biological Resources, Incheon 22689, Korea; itcha@korea.kr (I.-T.C.); dhjung529@gmail.com (D.-H.J.); 3 Personalized Diet Research Group, Korea Food Research Institute, Wanju 55365, Korea; youngdo98@kfri.re.kr (Y.-D.N.); slpark@kfri.re.kr (S.-L.P.); 4 Division of Bioengineering, Incheon National University, Incheon 22012, Korea; 5 Research Center for Bio Materials & Process Development, Incheon National University, Incheon 22012, Korea

**Keywords:** *Metabacillus*, marine mud, polyphasic taxonomy, isolation, carotenoid

## Abstract

The newly isolated strain KIGAM252^T^ was found to be facultatively anaerobic, Gram-stain-positive, spore-forming, and rod-shaped. They grew at 10–45 °C, pH 6.0–10.0, and were able to tolerate up to 6% NaCl in the growth medium. Phylogenetic analysis indicated that the KIGAM252^T^ strain was related to the genus *Metabacillus*. The cell membrane fatty acid composition of strain KIGAM252^T^ included C_15:0_ anteiso and C_15:0_ iso (25.6%) as the major fatty acids, and menaquinone 7 was the predominant isoprenoid quinone. The major polar lipids were diphosphatidylglycerol and phosphatidylglycerol. The size of the whole genome was 4.30 Mbp, and the G + C content of the DNA was 43.8%. Average nucleotide and amino acid identity and in silico DNA-DNA hybridization values were below the species delineation threshold. Pan-genomic analysis revealed that 15.8% of all genes present in strain KIGAM252^T^ was unique to the strain. The analysis of the secondary biosynthetic pathway predicted the carotenoid synthetic gene cluster in the strain KIGAM252^T^. Based on these current polyphasic taxonomic data, strain KIGAM252^T^ represents a novel species of the genus *Metabacillus* that produces carotenoids, for which we propose the name *Metabacillus flavus* sp. nov. The type of strain was KIGAM252^T^ (=KCTC 43261^T^ = JCM 34406^T^).

## 1. Introduction

The genus *Metabacillus* of the *Bacillaceae* family was first proposed by Patel and Gupta (2020). Most strains of the genus *Metabacillus* originally belonged to the genus *Bacillus*. Based on the overall genetic diversity, phylogenomic, and comparative genomic approaches, *Bacillus* species were proposed to be reclassified into six novel genera (*Peribacillus* gen. nov., *Cytobacillus* gen. nov., *Mesobacillus* gen. nov., *Neobacillus* gen. nov., *Alkalihalobacillus* gen. nov., and *Metabacillus* gen. nov.) [1]. All previously reported species of the genus *Metabacillus* are described as rod-shaped, Gram-stain-positive or Gram variable, and grow aerobically [1]. Recently, 14 species (*M. fastidiosus*, *M. indicus*, *M. herbersteinensis*, *M. litoralis*, *M. idriensis*, *M. niabensis*, *M. galliciensis*, *M. halosaccharovorans*, *M. crassostreae*, *M. endolithicus*, *M. malikii*, *M. mangrovi*, *M. iocasae*, and *M. lacus*) have been transferred from the genus *Bacillus* to the genus *Metabacillus* [2,3,4,5,6,7,8,9,10,11,12,13,14,15]. Only two species, *M. sediminilitoris* and *M. elymi* isolated from tidal sediments and plants native to the Dokdo Island were initially proposed as novel species of the genus *Metabacillus* [16,17]. The strain KIGAM252^T^ isolated in our study is a novel species of the genus *Metabacillus*, which can guide the future research of this genus and add to its taxonomic diversity.

Carotenoids are a large and diverse class of natural compounds comprising up to 700 structures produced by plants, fungi, and bacteria [18]. All carotenoids are derived from a precursor of isoprenoids, such as isopentenyl pyrophosphate (IPP) and its isomer dimethylallyl pyrophosphate (DMAPP), which is a C_5_ compound based on a carbon backbone, by mean of carotenoid biosynthesis [19]. In most bacteria, IPP and DMAPP participate in elongation reactions leading to the C_15_ (farnesyl pyrophosphate, FPP) and C_20_ (geranylgeranyl pyrophosphate, GGPP) carotenoid precursors [20]. Thus, microbial sources of carotenoids can produce a diverse range of carotenoids, such as those having C_30_, C_40_, and C_50_ backbones [21]. Among the currently known carotenoid-producing microbial sources, a wide range of colors, such as red, orange, yellow, brown, and black, can be obtained from *Bacillus* sp. alone [22,23]. The presence of varieties of glycosylated and/or esterified carotenoid derivatives, known as apo-carotenoids, has also been reported [24]. This diversity of pigments obtained from the *Bacillus* species is commercially attractive and has great potential for the development of microbial carotenoid production. In addition, the C_30_ carotenoid biosynthesis pathway has been established for *M. indicus* (originally described as *Bacillus indicus*) and *Cytobacillus firmus* (originally described as *Bacillus firmus*), and their carotenoid products have been identified [24,25,26]. In this study, we confirmed the synthetic pathways for the carotenogenic gene cluster and hypothesized the synthetic carotenoid that could potentially be produced by the strain KIGAM252^T^.

Marine mud is sediment that is generally found at the bottom of the sea. Since the Challenger expedition (1872–1876), there has been increased interest in the biology of microorganisms in the marine environment [27]. The marine environment has high hydraulic pressure and low temperature. Therefore, microorganisms isolated from marine mud may have particular characteristics that enable them to live in harsh environments. Various types of bacteria characterized as alkaliphiles, thermopiles, psychrophiles, and halophiles have been isolated in the marine environment [28,29]. Consequently, there is growing interest in the industrial use of microbes isolated from marine environments [30,31,32]. During the investigation of the diversity and characteristics of mud samples obtained from marine mud in the Uljin, we isolated a novel strain, which we designated as KIGAM252^T^, and considered it to belong to the genus *Metabacillus*. In this study, we performed a polyphasic taxonomy approach for physiological, chemotaxonomic, phylogenetic, and comparative genomic characterization of the isolate and proposed that the strain KIGAM252^T^ should be classified as a novel species of the genus *Metabacillus*, for which we propose the name *Metabacillus flavus* sp. nov.

## 2. Materials and Methods

### 2.1. Bacterial Strain Isolation and Cultivation

Strain KIGAM252^T^ was isolated from a marine mud sample obtained at a depth below 194 m from the Uljin area (36°42′21.8″ N, 129°35′0.7″ E), Republic of Korea, in March 2020. The obtained mud samples (0.5 g) were suspended in 5 mL of 2% NaCl and vortexed. Using the sample solution (0.5 mL), serial dilution was performed in fresh marine broth 2216 (BD, Franklin Lakes, NJ, USA). An aliquot (100 µL) of the diluted samples was spread onto marine broth containing 1.5% (*w/v*) agar. Each plate was incubated at different temperatures of 20 and 30 °C for 2 weeks, respectively, following which colonies with different colors were selected and repeatedly sub-cultured to the new marine agar (MA) at least thrice to obtain pure colonies. Finally, one slightly yellow colony of the species isolated from the MA agar plate was selected and designated as strain KIGAM252^T^ and stored at −80 °C in a 20% (*w/v*) glycerol stock solution for preservation.

### 2.2. DNA Extraction and Phylogenetic Analysis

Genomic DNA was extracted from strain KIGAM252^T^ using the HiYield^TM^ Genomic DNA Mini Kit (RBC Bioscience, New Taipei City, Taiwan). The partial 16S rRNA gene was amplified by polymerase chain reaction (PCR) using universal bacterial primers 27F, 337F, 785F, 800R, and 1492R [33]. Then, the PCR products were purified using the QIAquick PCR purification kit (Qiagen, Hilden, Germany). The purified PCR products were sent to Macrogen Co., Ltd. (Seoul, Korea) for sequence analysis. The sequenced products were assembled using a method described in a previous publication [34] using the SeqMan^TM^ *II* expert sequence analysis software [35]. Based on the EzBioCloud 16S-based ID (https://www.ezbiocloud.net/identify, 11 February 2021), sequences of the type strains closely related to strain KIGAM252^T^ were retrieved. The reference strain, *M. mangrovi* AK61^T^, was purchased from the Japan Collection of Microorganisms (JCM), and *M. indicus* LMG 22858^T^ was purchased from the Deutsche Sammlung von Mikroorganismen und Zellkulturen (DSMZ) and *M. idriensis* SMC 4352-2^T^ was purchased from the Korean Culture Center of Microorganisms (KCCM) to evaluate and compare their phenotypic properties and perform comparative analyses.

The taxonomic similarity of the resultant 16S rRNA gene sequence of strain KIGAM252^T^ and the closely related taxa was examined using the EzTaxon-e server (http://www.ezbiocloud.net/eztaxon/, 11 February 2021) [36]. Multiple sequence alignments were conducted using the Clustal W multiple sequence alignment program in the BioEdit 7.2.6.1 software [37,38]. Phylogenetic trees were constructed by phylogeny to build the application in MEGA 7.0 [39], based on the 16S rRNA gene sequence of KIGAM252^T^ and closely related taxa. Using the three algorithms, maximum-likelihood (ML), neighbor-joining (NJ), and maximum-parsimony (MP) [40,41,42], the sequence relatedness among strain KIGAM252^T^ and the other related taxa were calculated. The tests for phylogeny were conducted using the bootstrap method, and the bootstrap values were set to 1000 replicates. The Kimura two-parameter model was applied to calculate evolutionary distances [43]. *Lysinibacillus boronitolerans* 10a^T^ was used as the outgroup taxon.

### 2.3. Phenotypic Characterization

All experiments were performed on cultures grown at 30 °C in tryptic soy agar (TSA) and tryptic soy broth (TSB) (BD, Franklin Lakes, NJ, USA). The cell morphology of strain KIGAM252^T^ was examined by light microscopy (model CX 23; Olympus, Tokyo, Japan) and transmission electron microscopy (LIBRA 120; Carl Zeiss, Oberkochen, Germany). Cell motility tests were performed on 0.5% semi-solid TSB agar test tubes by stabbing inoculation [44]. Gram staining was performed using a Gram stain kit (BioWORLD, Dublin, OH, USA) according to the manufacturer’s instructions. To assess the cell growth at various salinities, cells were incubated in TSB media with NaCl concentrations ranging from 0% to 12% (*w/v*) at increments of 1% for 3 days at 30 °C and 180 rpm. The pH range for growth was assessed by growing the cells for 3 days on TSB media with increments of 1.0 pH unit from pH 4.0 to 12.0 at 30 °C and 180 rpm using the following buffer solutions: 100 mM CH_3_COOH/CH_3_COONa buffer (pH 4.0–6.0), 100 mM NaH_2_PO_4_/Na_2_HPO_4_ buffer (pH 7.0–8.0), 100 mM NaHCO_3_/Na_2_CO_3_ buffer (pH 9.0–10.0), and 100 mM Na_2_CO_3_/NaOH buffer (pH 11.0–12.0). The cells were cultured on TSA plates and incubated at 4, 10, 15, 20, 25, 30, 35, 37, 40, 45, 50, and 55 °C to determine the range of growth temperature. Tests for hydrolysis of casein, gelatin, starch, and Tween 20, 40, and 80 were performed on TSA plates according to a method described in a previous publication [45]. Catalase and oxidase tests were performed using 3% (*v/v*) H_2_O_2_ and 1% tetramethyl-p-phenylenediamine. Anaerobic growth was determined using the GasPak^TM^ EZ anaerobic gas generating pouch system with an indicator (BD, Franklin Lakes, NJ, USA) on TSA plates incubated for 2 weeks at 30 °C. The methyl red and Voges–Proskauer tests were conducted on MR-VP medium (BD, Franklin Lakes, NJ, USA) with methyl red solution and 5% α-naphthol solution. H_2_S formation was monitored on TSB media containing 0.5% (*w/v*) sodium thiosulfate with lead acetate paper. Antibiotic susceptibility tests were conducted on a TSA plate at 30 °C for 2 weeks using the paper disc method [46]. The antibiotics used were as follows (µg per disc): ampicillin (10 µg), anisomycin (50 µg), cephalothin (30 µg), erythromycin (25 µg), gentamicin (30 µg), kanamycin (30 µg), lincomycin (15 µg), neomycin (30 µg), norfloxacin (20 µg), novobiocin (10 µg), penicillin G (20 UI), streptomycin (50 µg), and tetracycline (30 µg). Other phenotypic and biochemical properties were examined using API 20NE, API^®^ ZYM, and API 50CH test kits according to the manufacturer’s instructions (bioMérieux, Marcy-l’Étoile, France).

### 2.4. Chemotaxonomic Characterization

Strain KIGAM252^T^ and closely related species (*M.* *mangrovi* AK61^T^, *M. indicus* LMG 22858^T^, and *M. idriensis* SMC 4352-2^T^) were cultivated on TSA at 30 °C for 2 days to analyze fatty acids. The analysis of cellular fatty acids was carried out by first subjecting the cells to saponification and methylation and then extracting the cellular fatty acids according to a method described in a previous publication using 6890 gas chromatography (Agilent, Santa Clara, CA, USA) and a cross-linked methyl siloxane column (HP-1; A30 m × 0.320 mm × 0.25 µm) [47]. Fatty acids were identified and quantified based on data from the TSBA6 database using the Sherlock MIS Software 6.2 [48]. Cells of strain KIGAM252^T^ were cultivated on TSB at 30 °C for 2 days for isoprenoid quinone and polar lipid profile analyses. The cells were freeze-dried, and the isoprenoid quinones were extracted according to the method described in the previous publication and separated by a YL9100 high-performance liquid chromatography (HPLC) system (Younglin, Anyang, Korea) [49]. The polar lipids were extracted and separated using the method described in a previous publication [50]. Polar lipid profiles were determined by subjecting the cell of strain KIGAM252^T^ to freeze-drying and then performing two-dimensional thin-layer chromatography (TLC) using 10 × 10 cm silica gel 60 F254 (Merck, Branchburg, NJ, USA) and detected using molybdophosphoric acid, Zinzadze’s reagent, and α-naphthol reagent.

### 2.5. Whole-Genome Sequencing and Verification of Authenticity of the Genome Assembly

Genomic DNA was extracted as described previously. The whole-genome sequence of strain KIGAM252^T^ was obtained and sequenced using a Pacific Biosciences RS II instrument with P6-C4 chemistry. De novo genome assembly was performed using Flye assembler 2.7 software with default parameters in PacBio SMRT Analysis v. 2.3.0 [51].

The authenticity and contamination check of the genome of strain KIGAM252^T^ was conducted according to the proposed minimal standards of using the prokaryote genome database [52]. The authenticity of strain KIGAM252^T^ was checked using 16S rRNA gene sequences obtained based on conventional Sanger sequencing and whole-genome sequencing results as described previously. The GenBank accession numbers of the 16S rRNA gene sequence and whole-genome sequences of strain KIGAM252^T^ were MT804551 and JAGVRK010000000, respectively. To verify the contamination in the genome assembly of strain KIGAM252^T^, the ContEst16S algorithm was used to assess each sequence (https://www.ezbiocloud.net/tools/contest16s, 11 February 2021) [53].

### 2.6. Genome Annotation and Phylogenomic and Comparative Genomic Analysis

In silico genome annotation of the strain KIGAM252^T^ was conducted by Rapid Annotations using Subsystems Technology (RAST) server (http://rast.nmpdr.org/, 21 April 2021) with the RASTtk pipeline [54,55]. Clusters of Orthologous Groups (COG) category analysis was performed by EggNOG v5.0 based on a search of predicted homologous genes [56,57]. Comparative genomic analysis was performed by obtaining full genome sequence data from the National Center for Biotechnology Information (NCBI) genome database (http://www.ncbi.nlm.nih.gov/genome/, 16 September 2021) for all species of the genus *Metabacillus*, namely *M. mangrovi* AK61^T^ (WMIB00000000), *M. indicus* LMG 22858^T^ (JNVC00000000), *M. idriensis* SMC 4352-2^T^ (WKKF00000000), *M. crassostreae* DSM 25387^T^ (JAFBDU010000001), *M. fastidiosus* NBRC 101226^T^ (BCVG01000001), *M. halosaccharovorans* DSM 25387^T^ (KV917371), *M.s iocasae* DSM 104297^T^ (JAFBFC010000001), *M. lacus* AK74^T^ (WKKI01000001), *M. litoralis* SW-211^T^ (VOQF01000001), *M. niabensis* 4T19^T^ (CADEPK010000001), and *M. sediminilitoris* DSL-17^T^ (CP046266). The secondary metabolite biosynthetic gene clusters were identified using antiSMASH 6.0 software with strict detection criteria and by additional features derived using various algorithms, including KnownClusterBlast, ClusterBlast, SubClusterBlast, ActiveSiteFinder, and RREFinder (https://antismash.secondarymetabolites.org/, 21 April 2021) [58]. Ortho-average nucleotide identity (OrthoANI) and average amino acid identity (AAI) values among the strain KIGAM252^T^ and other species of the genus *Metabacillus* were calculated using the OAT software and EzAAI tool [59,60] to analyze genomic relatedness, respectively. In silico DNA-DNA hybridization (*is*DDH) values were calculated using the Genome-to-Genome Distance Calculator program (GGDC 2.1; http://ggdc.dsmz.de/distcalc2.php, 16 September 2021) [61] with recommended formula 2 based on DNA-DNA hybridization between KIGAM252^T^ and other species of the genus *Metabacillus* [62]. The intergenomic relatedness was compared to that of the genus *Metabacillus* using the Type Strain Genome Server (TYGS) (http://tygs.dsmz.de/, 16 September 2021). A phylogenomic tree was constructed with branch support via FastME 2.1.4, including SPR post-processing using Genome Blast Distance Phylogeny (GBDP) distances, with the numbers above the branches being pseudo-bootstrap support values of GBDP based on 100 replications [63].

### 2.7. Pan-Genomic Analysis

Pan-genome was constructed using the Bacterial Pan Genome Analysis (BPGA) software [64]. All genomes of the genus *Metabacillus* and strain KIGAM252^T^ were defined as core (conserved for all strains), accessory (shared by more than two species but not core), and unique (strain-specific) genes. The pan-genome function and pathway analyses were performed based on data from the Database of COGs (https://www.ncbi.nlm.nih.gov/research/cog/, 16 September 2021) and the Kyoto Encyclopedia of Genes and Genomes (KEGG) database (https://www.genome.jp/kegg/, 16 September 2021) [65] for representative sequences of all orthologous gene families, and a comparative functional analysis of core, accessory, and unique genes were thus performed. For clustering COG and KEGG pan-genome orthologous groups (POGs), the USEARCH algorithm was used with a 50% sequence identity cut-off value. All the results of the plotting data in this analysis were visualized using gnuplot 4.6.6. Multiple sequence alignment performed by Multiple Sequence Comparison by Log-Expectation (MUSCLE) [52] was used to concatenate core genes to generate a phylogenetic tree using the NJ method with 1000 replications of bootstrap values [41].

### 2.8. Pigment Extraction and Analysis

A concentrated bacterial cell suspension was sonicated for 5 min and centrifuged for 5 min at 12,000× *g*. The supernatant was lyophilized and treated with acetone/methanol (7:3, *v/v*). The collected cells were treated with an equal volume of organic solvent, and the suspension was incubated at 37 °C and 180 rpm for 2 h in dark conditions to sufficiently decolorate. The extracts were centrifuged for 15 min at 12,000× *g*, and the upper organic layers of the extracts were pooled and evaporated using smart evaporator C1 (BioChromato, San Diego, CA, USA) at 40 °C and re-dissolved in methanol/dichloromethane (5:5, *v/v*). The obtained extracts were subsequently filtered through a 0.2 μm pore size nylon syringe filter (GVS Korea Ltd., Namyangju, Korea), and finally withdrew the crude carotenoid extracts.

The maximum absorbance spectra of the crude carotenoid extracts were measured using a UV-1280 Shimadzu UV-Visible spectrophotometer (Shimadzu, Kyoto, Japan). The crude extracted pigments were separated and detected using a YL9100 plus HPLC system equipped with a YL9160 photodiode array (PDA) detector (Youngin Chromass, Anyang, Korea). Injections (20 μL) were made, and separations were performed on a C_30_ (5 μm, 250 mm × 4.6 mm i.d.) reverse phase (RP) column (YMC Inc. Wilmington, DE, USA). The mobile phase was a gradient eluent containing methanol/water (92:8, *v/v*) with 10 mM ammonium acetate (solvent A) and 100% *tert*-butyl methyl ether (solvent B). Carotenoid extracts were eluted with a gradient of 90% solvent A and 10% solvent B for 20 min. A linear gradient was then initiated to reach 83% solvent A and 17% solvent B at 29 min. Subsequently, a sharply linear gradient was initiated for the elute to reach 30% solvent A and 70% solvent B at 35 min. Finally, a linear gradient was initiated for the elute to reach 25% solvent A and 75% solvent B at 42 min. The column was equilibrated and returned to the initial conditions for over 30 min. A flow rate of 1 mL min^−1^ was employed, and the profiles were recorded continuously with a PDA detector at 200–600 nm. Identification was performed based on spectral comparison using previously reported methods [24,66].

## 3. Results and Discussion

### 3.1. Morphological, Physiological, and Biochemical Characterization and Discrimination

Cells of the novel strain KIGAM252^T^ were Gram-stain-positive, non-motile, spore-forming, and rod-shaped, having a width of 1.5–1.6 µm and a length of 3.9–4.7 µm (Figure S1). Colonies were pale-yellow in color, circular, and flattened as observed on TSA incubated at 30 °C for 2 days. Growth of strain KIGAM252^T^ occurred at 10–45 °C (optimum, 30 °C) and pH 6.0–10.0 (optimum, pH 7.0). In particular, strain KIGAM252^T^ can grow and survive at NaCl concentrations of up to 6% (*w/v*) (optimal growth at 3% NaCl). This is a rather high tolerance for NaCl compared to that of other reference strains, maybe because of the strain KIGAM252^T^ being isolated from marine mud. Strain KIGAM252^T^ hydrolyzed casein and gelatin but did not hydrolyze starch or Tween 20, 40, and 80. Positive reactions were detected for catalase and oxidase tests. Strain KIGAM252^T^ was observed to grow under anaerobic conditions. The results of the methyl red and the Voges–Proskauer tests were negative, while the H_2_S formation showed positive results. Strain KIGAM252^T^ was resistant to ampicillin, anisomycin, cephalothin, lincomycin, and penicillin G. Whereas strain KIGAM252^T^ was susceptible to erythromycin, gentamicin, kanamycin, norfloxacin, novobiocin, neomycin, streptomycin, and tetracycline. The detailed characteristics of strain KIGAM252^T^ and related species in the API 20NE, API^®^ ZYM, and API 50CH tests are shown in Table 1.

### 3.2. Chemotaxonomic Characterization

The major fatty acid profile of strain KIGAM252^T^ showed C_15:0_ anteiso (35.7%), C_15:0_ iso (25.6%), C_16:0_ iso (9.8%), and C_17:0_ (8.1%) to be the most prevalent fatty acids. These results were similar to those of closely related type strains of the genus *Metabacillus* (Table 2). The isoprenoid quinone analysis showed that the strain KIGAM252^T^ had MK-7 as the predominant isoprenoid quinone. In the polar lipid profiling of strain KIGAM252^T^, diphosphatidylglycerol (DPG) and phosphatidylglycerol (PG) were identified as major polar lipids, while phosphatidylglycolipids (PGL), aminophospholipid (APL), glycolipids (GL), phospholipids (PL), and some unidentified lipids (L) were found to have minor distributions (Figure S2).

### 3.3. Whole-Genome Sequencing and Verification of Authenticity of the Genome Assembly

The genome sequence of the strain KIGAM252^T^ comprised two complete chromosomes; one is large (4,026,853 bp) and the other is small (5635 bp). The total genome size and G + C content of the DNA were 4,302,488 bp and 43.8%, respectively. Strain KIGAM252^T^ was predicted to harbor 4092 genes, including 3898 coding genes, 128 RNA genes, and 66 pseudo-genes. The numbers of rRNAs, tRNAs, and ncRNAs were 34, 89, and 5, respectively. More detailed general genomic features of KIGAM252^T^ are presented in Table 3.

The comparison of 16S rRNA gene sequences obtained from whole-genome sequencing and conventional Sanger sequencing showed 100% similarity for sequences of strain KIGAM252^T^ obtained by both methods. The authenticity of the genome of strain KIGAM252^T^ was thus verified, as the genome sequences were not found to be contaminated.

### 3.4. 16S rRNA Gene Phylogeny

**Figure 1:**
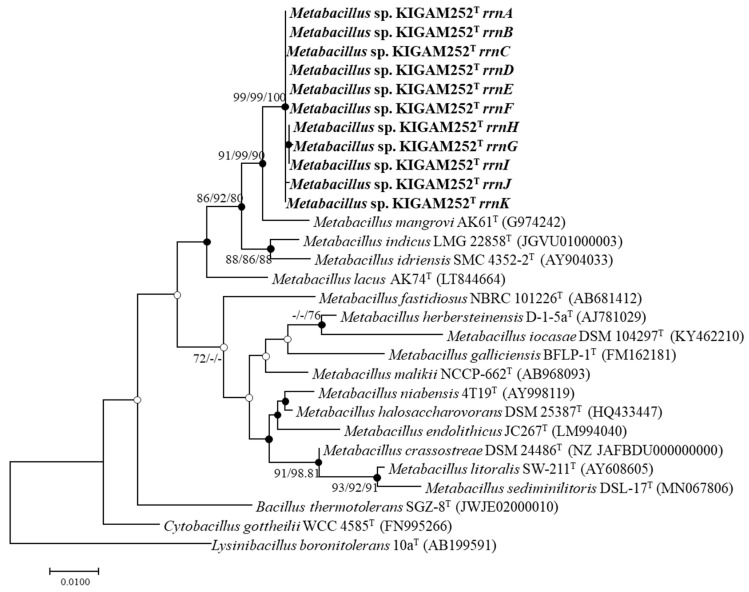
Maximum likelihood (ML) phylogenetic tree of strain KIGAM252^T^ and related type strains of *Metabacillus* species based on the 16S rRNA gene sequences. The closed circles represent nodes recovered by both the neighbor-joining (NJ) and maximum parsimony (MP) algorithms; the open circles represent nodes recovered by either NJ or MP. The numbers on the nodes indicate the bootstrap values (>70%) calculated using the ML/NJ/MP probabilities. Bar, 0.01 changes per nucleotide.

A total of 11 16S rRNA gene sequences of strain KIGAM252^T^ (1539 bp) were obtained by whole-genome sequencing, as mentioned above. In the phylogenetic analysis, strain KIGAM252^T^ and other closely related species showed 98.4% 16S rRNA gene sequence similarity (Table S1). The close phylogenetic relatives with the most similarities were *M. mangrovi* AK61^T^ (98.4%), *M. indicus* LMG 22858^T^ (97.6%), and *M. idriensis* SMC 4352-2^T^ (97.1%). Furthermore, according to the phylogenetic tree based on 16S rRNA gene sequences, the closest clustered species with strain KIGAM252^T^ was *M. mangrovi* AK61^T^ (91, 99, and 90 in ML, NJ, and MP trees, respectively). Strain KIGAM252^T^ and *M. mangrovi* AK61^T^ clustered with *M. indicus* LMG 22858^T^ and *M. idriensis* SMC 4352-2^T^ (86, 92, and 80 in ML, NJ, and MP trees, respectively) (Figure 1). According to the phylogenetic analysis, strain KIGAM252^T^ was closely related to the other *Metabacillus* species mentioned above, but the entire phylogenetic tree also showed that most of the type strains of the genus *Metabacillus* were distinct from strain KIGAM252^T^ [67]. These results suggest that strain KIGAM252^T^ represents a novel species in the genus *Metabacillus*.

### 3.5. Genome-Derived Features and Comparative Genomic Analysis

The COG analysis showed a total of 3656 genes to be present in strain KIGAM252^T^, and 2268 (62.0%) genes associated with the 18 general COG functional categories were classified as functional genes, excluding those classified as functional unknown (S). The most abundant predicted genes of strain KIGAM252^T^ belonged to the COG categories of amino acid transport and metabolism (E, 284, 7.8%), transcription (K, 228, 6.2%), carbohydrate transport and metabolism (G, 191, 5.2%), energy production and conversion (C, 178, 4.9%), and inorganic ion transport and metabolism (P, 175, 4.8%) (Table S2).

Biosynthetic gene clusters (BGCs) predicted by antiSMASH showed that the genomes of strain KIGAM252^T^ and other *Metabacillus* species had 4–10 BGCs responsible for the secondary metabolites, including the genes for terpenes, T3PKS, siderophores, lassopeptides, and other biosynthetic genes (LAP & RiPP-like, NRPS, and lanthiopeptide genes) (Table S3). Strain KIGAM252^T^ possesses six BGCs (two terpene genes, and T3PKS, siderophore, lassopeptide, and LAP & RiPP-like genes). However, only one terpene and BGC and lassopeptide BGC shared 66.0% similarity in two known pathways with the carotenoid BGC from *Halobacillus halophilus* DSM 2266^T^ (GenBank: FJ040212) [68] and 80.0% similarity with the paeninodin BGC from *Paenibacillus dendritiformis* C454^T^ (GenBank: AHKH01000064) [69] (Table S4). The other four BGCs in strain KIGAM252^T^ shared no similarity with already known pathways. Although the functions of the four BGCs cannot be predicted accurately, they are likely to produce novel bioactive compounds.

Among the BGCs with predicted similarity was already known pathways in KIGAM252^T^, the carotenoid-associated terpene BGC was detected in all species of the genus *Metabacillus*, but all clustering patterns of the genus *Metabacillus* were not equivalent. In addition, the most known clusters that shared the highest similarity with each terpene BGC of the genus *Metabacillus* were identified to be different. The biosynthetic pathway for carotenoids in strain KIGAM252^T^ showed that the gene for squalene/phytoene synthase family protein was the core gene, and genes for three phytoene desaturases and acyltransferase were additional genes. According to a previous report on C_30_ carotenoid gene annotation and functional assignment in the *Bacillus* species, various *crtN* genes belonging to the *crtI* gene family were found in *M. indicus* HU36 and *Cytobacillus firmus* GB1 [70]. In addition, glycosyl-transferase, acyltransferase, and carboxyltransferase, which modify carotenoid structures, were found in the two species. A comparison with other species of the genus *Metabacillus* showed strain KIGAM252^T^ to possess a C_30_ carotenoid biosynthetic gene cluster similar to that present in *M. indicus* HU36. The organization of genes associated with the carotenoid biosynthetic pathway in both *Metabacillus* species is shown by nucleotide-based genetic localization (Figure S3). Each gene has been shown previously to be annotated as diapophytoene synthase (*crtM*), 4,4′ -diapophytoene desaturase (*crtNa*), 4,4′ -diapophytoene-ketolase (*crtNb*), 4,4′ -diapophytoene aldehyde oxidase (*crtNc*), and acyltransferase (*AT*), respectively [70]. The sequence identities of each gene between strain KIGAM252^T^ and *M. indicus* HU36 were 47.7–66.4%, and the amino acid identities were 63.2–69.5%, respectively (Table S5). However, the amino acid identity of *AT* was significantly lower than that of other genes. Diapophytoene synthase (*crtM*) is predicted to have evolutionary relationships with bacterial phytoene synthase (*crtB*) [70]. In addition, 4,4′ -diapophytoene desaturase family genes (*crtNa*, *crtNb*, and *crtNc*) belong to the phytoene desaturase (*crtI*) family [71]. These four genes are related to the synthesis of the universal bacterial C_30_ carotenoid skeleton. Therefore, they can be conserved from generation to generation or even with interspecies variations. However, genes for glycosyl-transferase, acyltransferase, and carboxyltransferase are additional modification-associated genes involved in bacterial carotenoid synthesis. Therefore, these additional genes may be strain-specific or show high interspecies variation.

**Figure 2:**
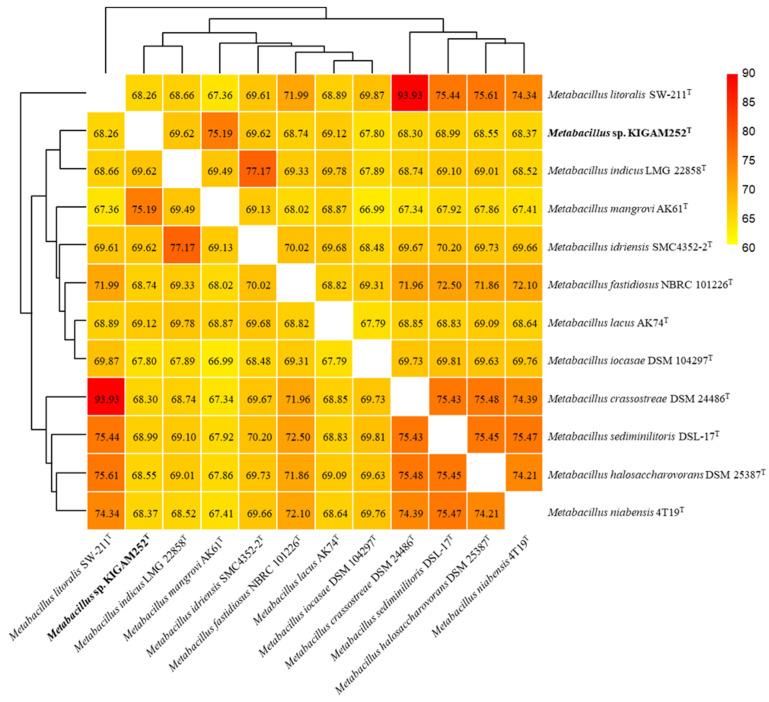
Heatmap based on OrthoANI values calculated for strain KIGAM252^T^ and other species of the genus *Metabacillus*; *M. mangrovi* AK61^T^, *M. indicus* LMG 22858^T^, *M. idriensis* SMC4352-2^T^, *M. lacus* AK74^T^, *M. sediminilitoris* DSL-17^T^, *M. fastidiosus* NBRC 101226^T^, *M. halosaccharovorans* DSM 25387^T^, *M. niabensis* 4T19^T^, *M. crassostreae* DSM 24486^T^, *M. litoralis* SW-2211^T^, and *M. iocasae* DSM 104297^T^. High OrthoANI value is indicated in red, whereas lower values are indicated in yellow.

The OrthoANI values computed by a comparison between strain KIGAM252^T^ and other species of the genus *Metabacillus* ranged from 67.8% to 75.2% (Figure 2). The highest OrthoANI value obtained by comparisons between strain KIGAM252^T^ and other *Metabacillus* species was 75.2%, as obtained by comparison with *M. mangrovi* AK61^T^, while comparisons with other species showed values under 70%. The AAI values were calculated to be ranging from 65.2% to 80.0% (Figure S4). The *is*DDH values obtained by comparisons between strain KIGAM252^T^ and other species of the genus *Metabacillus* did not exceed 30% (Table S6). According to the suggested cut-off values of OrthoANI, AAI, and *is*DDH for species delineation (less than 95%, 90%, and 70%, respectively) [40,45,54], the calculated values based on the genomic comparison results proposed that KIGAM252^T^ was distinguished from other previously reported *Metabacillus* species. In addition, the phylogenomic tree associated with intergenomic relatedness showed that the total 12 species of *Metabacillus*, including strain KIGAM252^T^, did not involve equivalent species and subspecies clustering (Figure S5). This result provided more evidence of distinctiveness between KIGAM252^T^ and other species of the genus *Metabacillus*.

### 3.6. Pan-Genomic Analysis of the Genus Metabacillus including Strain KIGAM252^T^

**Figure 3:**
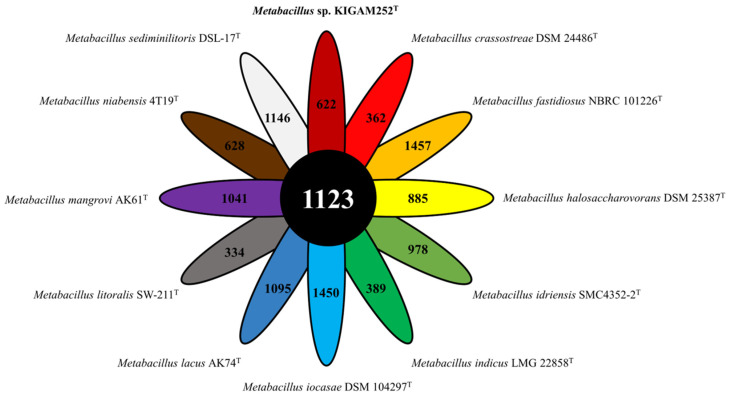
Flower plot indicating the core and strain-specific POGs of the novel strain KIGAM252^T^ and of other species of the genus *Metabacillus*; *M. mangrovi* AK61^T^, *M. indicus* LMG 22858^T^, *M. idriensis* SMC4352-2^T^, *M. lacus* AK74^T^, *M. sediminilitoris* DSL-17^T^, *M. fastidiosus* NBRC 101226^T^, *M. halosaccharovorans* DSM 25387^T^, *M. niabensis* 4T19^T^, *M. crassostreae* DSM 24486^T^, *M. litoralis* SW-2211^T^, and *M. iocasae* DSM 104297^T^.

The pan-genomic analysis showed that strain KIGAM252^T^ and 11 species of the genus *Metabacillus* had a total 18,494 of POGs: 1123 core POGs, 6984 accessory POGs, and 10,387 unique POGs (Figure S6). The genomes of all *Metabacillus* species show the core and accessory genes to constitute 24.0–30.3% and 32.9–66.5% of protein-coding genes, respectively (Table 4). In addition, strain KIGAM252^T^ had 622 (15.8%) unique POGs, and other species also had various numbers of strain-specific unique POGs (Figure 3). This observation suggests that all species analyzed by the pan-genome analysis were distinct. According to the COG database, strain KIGAM252^T^ had 221 unique POGs, and most POGs were related to Transcription (K, 11.4%), Carbohydrate transport and metabolism (G, 8.1%), Cell wall/membrane/envelope biogenesis (M, 7.1%), and Amino acid transport and metabolism (E, 6.6%) (Figure S7A). Such categorization showed a similar trend for all *Metabacillus* species (less than 3% difference) (Figure S7C). The categories of Replication, recombination, and repair (L) and Inorganic transport and metabolism (P) were enriched in strain KIGAM252^T^ (14.0% and 11.0%, respectively) compared to those in other *Metabacillus* species (6.1% and 5.7%, respectively). Strain KIGAM252^T^ had 5.3% POGs in the category of signal transduction mechanisms (T), consequently making this category more depleted than that in all species of *Metabacillus* (9.4%). According to the KEGG database, 159 POGs were confirmed as KEGG categorizations of unique genes, of which 97 POGs were defined as functional KEGG annotations (Table S7). Enrichment of the KEGG categories of the unique POGs in strain KIGAM252^T^ was predicted to be that of the metabolism (47.4%) and environmental information processing-related pathways (18.6%) (Figure S7B). Similar to the results of the COG categorization, most of the strain KIGAM252^T^-specific POGs were annotated to genes of carbohydrate metabolism (11.3%), amino acid metabolism (11.3%), and membrane transport (12.4%) subcategories. Various dehydrogenases, transferases, transaminases, kinases, deacetylases, mutases, ligases, and synthases of the carbohydrate and amino acid metabolism subcategories were annotated, and the phosphotransferase system (PTS), bacterial secretion system, and ABC transporters of the membrane transport subcategory were detected. In addition, the phylogenomic tree based on concatenated core POGs showed that the strain KIGAM252^T^ was distinct from the other *Metabacillus* species, as being clustered with *M. mangrovi* AK61^T^, and the two species clustered closely with *M. indicus* LMG 22858^T^ and *M. idriensis* SMC4352-2^T^ (Figure S8). The construction of the phylogenetic tree based on the 16S rRNA gene sequences showed the same tendency, which suggests that strain KIGAM252^T^ and *Metabacillus mangrovi* AK61^T^ are the closest evolutionary related species. According to the pan-genomic analysis, strain KIGAM252^T^ had more unique POGs and a distribution of unique genes distinguished from other *Metabacillus* species. Thus, the overall pan-genomic analysis confirmed that strain KIGAM252^T^ is a novel species in the genus *Metabacillus*.

### 3.7. Identification of Carotenoid in the Strain KIGAM252^T^

**Figure 4:**
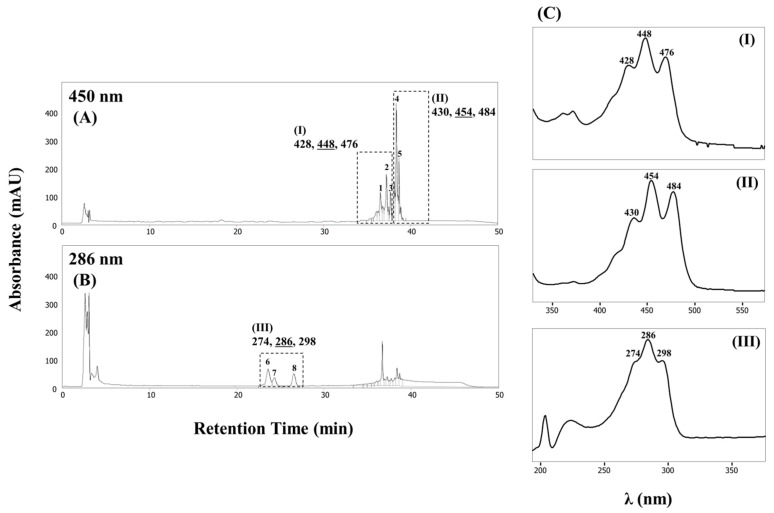
HPLC analysis of extracts from strain KIGAM252^T^. Chromatogram of carotenoid extracts recorded at 450 nm (**A**) and at 286 nm (**B**). UV/Vis spectra (**C**) grouped (**I**–**III**) according to their chromatographic peaks as shown in panels (**A**,**B**).

The yellow carotenoid formed by strain KIGAM252^T^ was extracted and subjected to HPLC-PDA analysis. The chromatographic profiles were obtained at 450 and 286 nm (Figure 4A,B). At 450 nm, the peaks could be categorized into two groups according to their absorbance spectra. Group I had a visible spectral maximum of 448 nm and contained predominant peaks 1–3, eluting between 36.54 and 37.70 min (Figure 4(C-I)). Group II contained predominant peaks 4 and 5, eluting between 38.38 and 38.68 min during which the chromatographic components all had a maximum absorption spectrum of 454 nm (Figure 4(C-II)). Chromatographic peaks 6–8 at 286 nm, eluting from 23.6 to 26.5 min represent group III. These chromatographic components all had a maximum absorption spectrum at 286 nm (Figure 4(C-III)). The major carotenoid products of strain KIGAM252^T^ were predicted to be apo-8′-phytoene, 1-glycosyl-apo-8′-lycopene, and their isomers, based on comparisons of the observed spectral properties with those previously reported for *Bacillus* carotenoids (Table S8) [24,70]. Although additional analyses will be necessary for accurate identification, the products of carotenoid of strain KIGAM252^T^ were highly similar to those of *M. indicus* HU36 [24,70]. Based on the results of this study and previous studies on this topic, we confirmed the tendency of carotenoid production in *Metabacillus* species, which can provide better insights into carotenoid production in microorganisms.

## 4. Conclusions

This study provides the first genome-based approach, including pan-genome analysis, conducted on the genus *Metabacillus*, and permits the classification of novel species, whose description is given below. In addition, carotenoid biosynthesis in strain KIGAM252^T^ was investigated using genomic comparison as previously reported. Carotenoid extracted from KIGAM252^T^ cell was confirmed using spectrometric and chromatographic analyses. Overall phenotypic and genome-based analyses have been performed to explore the possibilities of taxonomic distinctions compared with other *Metabacillus* species previously reported. Based on the results, we propose that strain KIGAM252^T^ represents the type strain of a novel species of the genus *Metabacillus* that produces carotenoids, for which we propose the name *Metabacillus flavus* sp. nov.

### Description of Metabacillus flavus sp. nov.

*Metabacillus flavus* (fla’vus. L. masc. adj. *flavus*, yellow, referring to the color of the colonies).

Cells are Gram-stain-positive, non-motile, spore-forming, rod-shaped cells with 1.5–1.6 µm in width by 3.9–4.7 µm in length. Colonies are pale-yellow color and circular and flatted on TSA incubated at 30 °C for 2 days. Growth occurs in the 10–45 °C (optimum, 30 °C) at 0–6% NaCl (optimum, 3%) at pH 6.0–10.0 (optimum, pH 7.0). The strain hydrolyzes of casein and gelatin but does not hydrolyze starch or Tweens 20, 40, and 80. The catalase and oxidase activities are positive and can grow under anaerobic conditions. The methyl-red and Voges–Proskauer tests are negative and H_2_S production is positive. In the API 20NE test, *β*-glucosidase, protease, and *β*-galactosidase activities are positive, but the reduction of nitrate to nitrite, reduction of nitrite to nitroxide, indole production, glucose fermentation, arginine dihydrolase, and urease activities are negative. In the API^®^ ZYM test, alkaline phosphatase, esterase lipase (C8), leucine arylamidase, trypsin, *α*-chymotrypsin, naphthol-AS-BI-phosphohydrolase, *α*-galactosidase, *β*-galactosidase, and *α*-glucosidase activities are positive, but esterase (C4), lipase (C14), valine arylamidase, cystine arylamidase, acid phosphatase, *β*-glucuronidase, *β*-glucosidase, *N*-acetyl-*β*-glucosaminidase, *α*-mannosidase, and *α*-fucosidase activities are negative. The following sole carbon and energy sources are used for growth in the API 50CH test, glycerol, D-galactose, D-glucose, D-fructose, D-mannitol, methyl-*α*-D-mannoside, methyl-*α*-D-glucoside, amygdalin, arbutin, esculin, salicin, D-cellobiose, D-maltose, sucrose, D-trehalose, D-melezitose, and D-raffinose. Major cellular fatty acids are C_15:0_ anteiso, C_15:0_ iso, C_16:0_ iso, and C_17:0_. Predominant isoprenoid quinone is MK-7, and the major polar lipids are DPG and PG. The C_30_ carotenoid 1-glycosyl-apo-8′-lycopene and its isomers are found in cell extracts. The NCBI GenBank accession numbers for the 16S rRNA gene sequence and complete genome are MT804551 and JAGVRK010000000, respectively. The G + C content of the DNA is 43.8% (complete genome sequences). The type strain, KIGAM252^T^ (=KCTC 43261^T^ =JCM 34406^T^), was isolated from marine mud in the Uljin area, Republic of Korea.

## Figures and Tables

**Table 1:** Differential characteristics between strain KIGAM252^T^ and the type strains of closely related species in the genus *Metabacillus* (Taxa: 1, Strain KIGAM252^T^; 2, *M. mangrovi* AK61^T^; 3, *M. indicus* LMG 22858^T^; 4, *M. idriensis* SMC 4352-2^T^). All of the species were negative for the hydrolysis of Tween 20, 40, and 80 and Voges-Proskauer test. All of the species gave positive results for *β*-glucosidase, protease, and *β*-galactosidase, but negative results for reduction of nitrate to nitrite, reduction of nitrite to nitroxide, indole production, glucose fermentation, arginine dihydrolase, and urease in the API 20NE test. All of the species gave positive results for alkaline phosphatase, esterase lipase (C8), leucine arylamidase, naphthol-AS-BI-phosphohydrolase, *α*-galactosidase, *β*-galactosidase, and *α*-glucosidase, but negative results for *β*-glucuronidase, *N*-acetyl-*β*-glucosaminidase, *α*-mannosidase, and *α*-fucosidase in the API^®^ ZYM test. All of the species gave positive results for glycerol, D-galactose, D-glucose, D-fructose, esculin, D-maltose, and D-raffinose, but negative results for erythritol, D-arabinose, L-arabinose, L-xylose, D-adonitol, methyl-*β*-D-xyloside, L-sorbose, L-rhamnose, dulcitol, xylitol, D-lyxose, D-tagatose, D-fucose, L-fucose, D-arabitol, L-arabitol, gluconate, and 5-ketogluconate in the API 50CH test.

Characteristic	1	2	3	4
Temperature range (°C) (optimum)	10–35 (30) *	15–42 ^a^	15–37 ^b^	15–45 ^c^
NaCl range (%, *w/v*) (optimum)	0–6 (3) *	0–3 ^a^	0–2 ^b^	0–3 ^c^
pH range for growth (optimum)	6.0–10.0 (7.0) *	6.0–9.0 ^a^	6.0–7.0 ^b^	5.5–9.5 ^c^
Catalase *	+	−	+	−
Oxidase *	+	−	+	+
Hydrolysis of *				
Casein	+	−	+	+
Gelatin	+	−	−	−
Starch	−	+	+	+
Methyl red *	−	−	+	−
H_2_S production *	+	−	−	−
Esterase (C4)	−	+	+	+
Lipase (C14)	−	+	−	−
Valine arylamidase	−	+	+	+
Cystine arylamidase	−	+	−	−
Trypsin	+	−	−	−
*α*-Chymotrypsin	+	−	+	+
Acid phosphatase	−	−	−	+
*β*-glucosidase	−	+	−	−
D-Ribose	+	−	−	+
D-Xylose	−	−	−	+
D-Mannose	−	−	+	+
Inositol	−	−	−	+
D-Mannitol	+	+	−	+
D-Sorbitol	−	−	−	+
Methyl-*α*-D-mannoside	+	−	+	−
Methyl-*α*-D-glucoside	+	−	+	+
*N*-acetylglucosamine	+	−	+	+
Amygdalin	+	−	+	+
Arbutin	+	−	−	+
Salicin	+	−	+	+
D-Cellobiose	+	−	+	−
D-Lactose	−	−	+	+
D-Melibiose	−	+	+	+
Sucrose	+	+	−	+
D-Trehalose	+	+	−	+
Inulin	−	+	+	−
D-Melezitose	+	−	−	+
Starch	−	+	+	+
Glycogen	−	+	+	+
Gentiobiose	−	−	+	−
D-Turanose	−	−	+	+
2-Ketogluconate	−	−	+	+

* Data from this study. ^a^ Data from [6]; ^b^ [12]; ^c^ [7].

**Table 2:** Cellular fatty acid contents (>1% of the total fatty acids) of strain KIGAM252^T^ and related species. Taxa: 1, Strain KIGAM252^T^; 2, *M.* *mangrovi* AK61^T^; 3, *M. indicus* LMG 22858^T^; 4, *M. idriensis* SMC 4352-2^T^. All data are from this study under the same condition.

Saturated	1	2	3	4
C_10:0_				
C_12:0_	0.47	0.10	0.04	0.16
C_14:0_	0.21	0.12	ND	0.15
C_16:0_	1.50	3.00	0.89	0.62
C_17:0_	8.14	9.49	11.02	3.55
C_18:0_	ND	ND	0.09	ND
**Unsaturated**				
C_16:1_ ω7c alcohol	ND	2.04	2.36	3.78
C_16:1_ ω11c	ND	5.75	8.71	0.73
C_18:1_ ω9c	ND	ND	0.22	ND
**Branched-chain fatty acid**				
C_11:0_ iso	ND	0.22	ND	0.09
C_13:0_ iso	ND	0.12	ND	ND
C_14:0_ iso	7.81	3.81	4.04	6.88
C_15:0_ iso	25.61	39.78	19.29	21.92
C_16:0_ iso	9.78	6.34	7.59	10.35
C_17:0_ iso	4.67	4.76	6.44	5.54
C_17:1_ iso ω10c	ND	2.16	2.41	0.28
C_18:0_ iso	ND	ND	0.20	0.32
C_19:0_ iso	ND	ND	0.08	ND
C_11:0_ anteiso	ND	0.18	ND	0.19
C_15:0_ anteiso	35.68	15.45	23.75	35.35
C_17:0_ anteiso	5.79	5.44	10.15	9.09
**Summed feature**				
3; C_16:1_ ω7c/C_16:1_ ω6c	ND	ND	0.17	ND
4; C_17:1_ iso /C_17:1_ anteiso B	ND	0.99	1.48	0.74

ND, not detected; TR, trace amount (<1%).

**Table 3:** General characteristics of the genome of strain KIGAM252^T^.

Attribute	Characteristics
Sequencing platforms	PacBio
Assembler	FLYE v. 2.7
Genome coverage	292.0×
Assembly status	Complete
Assembly size (bp)	4,302,488 bp
G + C content (%)	43.8
N50	4,026,853
Total contigs	2
Chromosome	2
G + C content (%)	43.78 (JAGVRK010000001) 52.19 (JAGVRK010000002)
Total genes	4092
Total CDS	3964
Coding genes	3898
Pseudo genes	66
RNAs	128
-rRNA genes (5S, 16S, 23S)	11, 11, 12
-tRNAs	89
-ncRNAs	5

**Table 4:** The fraction of core, accessory, and unique genes of strain KIGAM252^T^ and related *Metabacillus* species.

Organism	Genes	Core (%)	Accessory (%)	Unique (%)
*Metabacillus* sp. KIGAM252^T^	3919	28.7	55.5	15.9
*Metabacillus crassostreae* DSM 24486^T^	4416	25.4	66.4	8.2
*Metabacillus fastidiosus* NBRC 101226^T^	4163	27.0	38.0	35.0
*Metabacillus halosaccharovorans* DSM 25387^T^	4786	23.5	58.0	18.5
*Metabacillus idriensis* SMC4352-2^T^	4599	24.4	54.3	21.3
*Metabacillus indicus* LMG 22858^T^	3704	30.3	59.2	10.5
*Metabacillus iocasae* DSM 104297^T^	3834	29.3	32.9	37.8
*Metabacillus lacus* AK74^T^	3767	29.8	41.1	29.1
*Metabacillus litoralis* SW-211^T^	4350	25.8	66.5	7.7
*Metabacillus mangrovi* AK61^T^	3947	28.5	55.6	15.9
*Metabacillus niabensis* 4T19^T^	4546	24.7	52.4	22.9
*Metabacillus sediminilitoris* DSL-17^T^	4670	24.0	51.4	24.5

## Data Availability

The datasets for this study can be found in online repositories. The NCBI GenBank accession numbers for the 16S rRNA gene sequence and complete genome of strain KIGAM252^T^ are MT804551 (https://www.ncbi.nlm.nih.gov/nuccore/MT804551, 1 August 2020) and JAGVRK010000000 (https://www.ncbi.nlm.nih.gov/nuccore/JAGVRK000000000, 11 May 2021), respectively.

## Supplementary Materials

The following supporting information can be downloaded at: https://www.mdpi.com/article/10.3390/microorganisms10050979/s1, Figure S1: Transmission electron micrograph of negatively stained strain KIGAM252^T^. Strain KIGAM252^T^ was cultivated at 30 °C for two days in TSA. Bar, 0.5 µm; Figure S2: Thin-layer chromatograms of the polar lipids of strain KIGAM252^T^. The 1st-D developing agent, chloroform:methanol:water (65:25:4, *v*/*v*/*v*) and the 2nd-D agent, chloroform:acetic acid:methanol:water (80:15:12:4, *v*/*v*/*v*/*v*). The major polar lipids are diphosphatidylglycerol (DPG) and phosphatidylglycerol (PG). PGL, phosphatidylglycolipid; APL, aminophospholipid; PL, unidentified phospholipid; GL, unidentified glycolipid; L, unidentified lipid; Figure S3: Carotenoid biosynthetic gene cluster comparison between strain KIGAM252^T^ and *M. indicus* HU36 based on the nucleotide sequence similarities. The shaded pink areas between linear structures indicate homologous regions. The gene: *crtM*, 4,4′-diapophytoene synthase; *crtNa*, 4,4′-diapophytoene desaturase; *crtNb*, 4,4′-diapophytoene-ketolase; *crtNc*, 4,4′-diapophytoene aldehyde oxidase; *AT*, acyltransferase. The genes indicated by gray arrows represent those that have no relationship with carotenoid production.; Figure S4: Heatmap based on AAI values calculated for strainKIGAM252^T^ and other species of the genus *Metabacillus*; *M. mangrovi* AK61^T^, *M. indicus* LMG 22858^T^, *M. idriensis* SMC4352-2^T^, *M. lacus* AK74^T^, *M. sediminilitoris* DSL-17^T^, *M. fastidiosus* NBRC 101226^T^, *M. halosaccharovorans* DSM 25387^T^, *M. niabensis* 4T19^T^, *M. crassostreae* DSM 24486^T^, *M. litoralis* SW-2211^T^, and *M. iocasae* DSM 104297^T^. High AAI value is indicated in navy, whereas lower value is indicated in sky blue; Figure S5: Phylogenomic tree based on TYGS results showing the relationship between strain KIGAM252^T^ with related type strains in genus *Metabacillus*. The whole-genome sequence-based tree was inferred with FastME 2.1.6.1 [63] from GBDP distances. Calculated from genome sequences. The branch lengths are scaled in terms of GBDP distance formula d5. The numbers above branches are GBDP pseudo-bootstrap support values > 60 % from 100 replications, with average branch support of 98.7 %. Percent of genomic G+C content ranges between 33.8 and 46.6 %; genome size ranges from 3.9 to 5.6 Mb; The number of proteins coded by each genome ranges between 3907 and 5263; Figure S6: Pan-genome and clustering analysis of strain KIGAM252^T^ and other species of the genus *Metabacillus*. The boxplots of the pan (purple color) and core genomes (yellow color) are progressively increasing or decreasing by the number of genomes; Figure S7: COG and KEGG categorization of unique genes of strain KIGAM252^T^. The strain-specific functional unique POG annotation of strain KIGAM252^T^ using COG database (A) and The strain-specific functional unique POG annotation of strain KIGAM252^T^ using the KEGG database (B). Fraction of unique genes associated with different COG categories for strain KIGAM252^T^ and related strains of the genus *Metabacillus*; *M. mangrovi* AK61^T^, *M. indicus* LMG 22858^T^, *M. idriensis* SMC4352-2^T^, *M. lacus* AK74^T^, *M. sediminilitoris* DSL-17^T^, *M. fastidiosus* NBRC 101226^T^, *M. halosaccharovorans* DSM 25387^T^, *M. niabensis* 4T19^T^, *M. crassostreae* DSM 24486^T^, *M. litoralis* SW-2211^T^, and *M. iocasae* DSM 104297^T^ (C). One-letter abbreviations for the COG categories: J, translation, ribosomal structure and biogenesis; K, transcription; L, replication, recombination, and repair; B, chromatin structure and dynamics; D, cell cycle control, cell division, chromosome partitioning; V, defense mechanisms; T, signal transduction mechanisms; M, cell wall/membrane/envelope biogenesis; N, cell motility; U, intracellular trafficking, secretion, and vesicular transport; O, post-translational modification, protein turnover, and chaperones; C, energy production and conversion; G, carbohydrate transport and metabolism; E, amino acid transport and metabolism; F, nucleotide transport and metabolism; H, coenzyme transport and metabolism; I, lipid transport and metabolism; P, inorganic ion transport and metabolism; Q, secondary metabolites biosynthesis, transport; R, general function prediction only, and catabolism; S, function unknown; Figure S8: Phylogenomic tree using concatenated 347 core genes of strain KIGAM252^T^ and other species of the genus *Metabacillus*. The tree was constructed using the NJ method with 1000 bootstrap replications; Table S1: 16S rRNA gene similarities between the strain KIGAM252^T^ and related taxa in the genus *Metabacillus*; Table S2: COG categories of coding proteins in strain KIGAM252^T^ genome; Table S3: The number of predicted secondary metabolite biosynthetic gene clusters (BGCs) and distribution of BGCs of strain KIGAM252^T^ and *Metabacillus* species.; Table S4: Distribution of BGCs of strain KIGAM252^T^ and similar known pathways with strict detection criteria.; Table S5: Sequence similarities of carotenoid biosynthetic genes between strain KIGAM252^T^ and *M. indicus* HU36. All sequence identities were calculated by NCBI BLASTn and BLASTp (http://blast.ncbi.nlm.nih.gov/blast/, 27 July 2020) database; Table S6: In silico DDH (*is*DDH) values and G+C content differences between strain KIGAM252^T^ and closely related species of the genus *Metabacillus*; Table S7: Strain-specific POGs annotated and classified of strain KIGAM252^T^ using the KEGG database; Table S8: Carotenoid identification based on chromatographic and spectral properties of carotenoids detected in extracts of strain KIGAM252^T^. Electronic supplementary data are available with the online Supplementary Material.
