# Supplementary material for: Genomic and Physiological Characterization of Metabacillus flavus sp. nov., a Novel Carotenoid-Producing Bacilli Isolated from Korean Marine Mud
Source: Microorganisms. 2022 May 7;10(5):979. doi: 10.3390/microorganisms10050979 (PMC9146079; doi:10.3390/microorganisms10050979)
Supplement: Supplementary file 1 [file microorganisms-10-00979-s001.zip › microorganisms-1704712-supplementary.pdf]

Supplementary Materials

Genomic and Physiological Characterization of *Metabacillus flavus* sp. nov., a Novel Carotenoid-Producing *Bacilli* Isolated from Korean Marine Mud

Chi Young Hwang [1], Eui-Sang Cho [1], Deok Jun Yoon [1], In-Tae Cha [2], Dong-Hyun Jung [2], Young-Do Nam [3],
So-Lim Park [3], Seong-Il Lim [3,*] and Myung-Ji Seo [1,4,5,*]

1 Department of Bioengineering and Nano-Bioengineering, Incheon National University, Incheon 22012, Korea; hcyoung28@gmail.com (C.Y.H.); whdmltkd123@gmail.com (E.-S.C.);
deockjoon.yoon@gmail.com (D.J.Y.)
2 Microorganism Resources Division, National Institute of Biological Resources, Incheon 22689, Korea; itcha@korea.kr (I.-T.C.); dhjung529@gmail.com (D.-H.J.)
3 Personalized Diet Research Group, Korea Food Research Institute, Wanju 55365, Korea; youngdo98@kfri.re.kr (Y.-D.N.); slpark@kfri.re.kr (S.-L.P.)
4 Division of Bioengineering, Incheon National University, Incheon 22012, Korea
5 Research Center for Bio Materials & Process Development, Incheon National University,
Incheon 22012, Korea
* Correspondence: silim@kfri.re.kr (S.-I.L.); mjseo@inu.ac.kr (M.-J.S.)

Table S1. 16S rRNA gene similarities between the strain KIGAM252^T and related taxa in the genus *Metabacillus*.

axon	Accession no.	Similarity (%)
Metabacillus mangrovi AK61^T	HG974242	98.4
Metabacillus indicus LMG 22858^T	JGVU01000003	97.7
Metabacillus idriensis SMC 4352-2^T	AY904033	97.6
Metabacillus lacus AK74^T	LT844664	97.1
Metabacillus niabensis 4T19^T	AY998119	96.6
Metabacillus halosaccharovorans DSM 25387^T	HQ433447	96.6
Metabacillus herbersteinensis D-1-5-a^T	AJ781029	96.5
Metabacillus malikii NCCP-662^T	AB968093	96.3
Metabacillus endolithicus JC267^T	LM994040	95.9
Metabacillus crassostreae DSM 25387^T	HQ419276	95.9
Metabacillus elymi	KC414705	95.5
Metabacillus iocasae DSM 104297^T	KY462210	95.4
Metabacillus galliciensis BFLP-1^T	FM162181	95.4
Metabacillus sediminilitoris DSL-17^T	MN67806	95.3
Metabacillus fastidiosus NBRC 101226^T	AB681412	95.3
Metabacillus litoralis SW-211^T	AY608605	95.1

Table S2. COG categories of coding proteins in strain KIGAM252^T genome.

Code	Description	Count	%
J	Translation, ribosomal structure, and biogenesis	166	4.5
K	Transcription	228	6.2
L	Replication, recombination and repair	155	4.2
D	Cell cycle control, cell division, chromosome partitioning	34	0.9
V	Defense mechanisms	47	1.3
T	Signal transduction mechanisms	132	3.6
M	Cell wall/membrane/envelope biogenesis	164	4.5
N	Cell motility	43	1.2
U	Intracellular trafficking, secretion, and vesicular transport	42	1.2
O	Posttranslational modification, protein turnover, chaperones	103	2.8
C	Energy production and conversion	178	4.9
G	Carbohydrate transport and metabolism	191	5.2
E	Amino acid transport and metabolism	284	7.8
F	Nucleotide transport and metabolism	83	2.3
H	Coenzyme transport and metabolism	98	2.7
I	Lipid transport and metabolism	94	2.6
P	Inorganic ion transport and metabolism	175	4.8
Q	Secondary metabolites biosynthesis, transport, and catabolism	51	1.4
S	Function unknown	1388	38.0
Total		3656	100

Table S3. The number of predicted secondary metabolite biosynthetic gene clusters (BGCs) and distribution of BGCs of strain KIGAM252^T and *Metabacillus* species.

Query Genome	Terpene	T3PKS	Siderophore	Lassopeptide	Others	Total
**KIGAM252^T**	2	1	1	1	1	6
Metabacillus crassostreae DSM 24486^T	1	1	1	1	1	5
Metabacillus fastidiosus NBRC 101226^T	3	1	1	0	5	10
Metabacillus halosaccharovorans DSM 25387^T	3	1	1	1	2	8
Metabacillus idriensis SMC4352-2^T	3	1	1	0	1	6
Metabacillus indicus LMG 22858^T	2	1	1	1	0	5
Metabacillus iocasae DSM 104297^T	2	1	1	0	1	5
Metabacillus lacus AK74^T	2	1	1	0	0	4
Metabacillus litoralis SW-211^T	2	1	1	1	0	5
Metabacillus mangrovi AK61^T	2	1	1	0	1	5
Metabacillus niabensis 4T19^T	2	1	0	1	1	5
Metabacillus sediminilitoris DSL-17^T	2	1	2	1	1	7

Table S4. Distribution of BGCs of strain KIGAM252^T and similar known pathways with strict detection criteria.

Gene type	Product	Span (nt)	Core Gene Similarity (%)	Most Similar Biosynthetic Gene Cluster (BGC)	BGC Similarity (%)
Terpene	Carotenoid	1,152,256-1,171,168	53.3	*Halobacillus halophilus* DSM 2266^T	66
Lassopeptide	Paeninodin	2,471,073-2,494,928	55.3	*Paenibacillus dendritiformis* C454^T	80

Table S5. Sequence similarities of carotenoid biosynthetic genes between strain KIGAM252^T and *M. indicus* HU36. All sequence identities were calculated by NCBI BLASTn and BLASTp (http://blast.ncbi.nlm.nih.gov/blast/) database.

**Query Sequence**	**Reference Sequence**	**Gene**	**Sequence Identity (%)**	**Amino Acid Identity (%)**
KIGAM252^T	*Metabacillus indicus* HU36	*crtM*	64.6	63.5
		crtNa	66.4	69.5
		crtNb	61.9	63.2
		crtNc	47.7	65.3
		AT	52.8	NI

NI, no identified of similarity.

Table S6. *In silico* DDH (*is*DDH) values and G+C content differences between strain KIGAM252^T and closely related species of the genus *Metabacillus*.

**Query Genome**	**Reference Genome**	***is*DDH Value (%)**	**Model Confidence Interval (%)**	**G+C Content Difference (%)**
KIGAM252^T	*Metabacillus halosaccharovorans* DSM 25387^T	29.3	[26.9 - 31.8]	7.66
	Metabacillus crassostreae DSM 24486^T	27.0	[24.6 - 29.5]	10.0
	Metabacillus sediminilitoris DSL-17^T	26.7	[24.4 - 29.2]	7.83
	Metabacillus iocasae DSM 104297^T	24.9	[22.6 - 27.4]	6.18
	Metabacillus niabensis 4T19^T	23.2	[20.9 - 25.7]	8.25
	Metabacillus fastidiosus NBRC 101226^T	22.4	[20.2 - 24.9]	8.69
	Metabacillus lacus AK74^T	21.6	[19.4 - 24.0]	2.17
	Metabacillus idriensis SMC4352-2^T	21.4	[19.2 - 23.8]	3.07
	Metabacillus indicus LMG 2285^T	21.4	[19.2 - 23.8]	0.57
	Metabacillus litoralis SW-211^T	21.2	[19.0 - 23.6]	10.0
	Metabacillus mangrovi AK61^T	19.9	[17.7 - 22.3]	2.85

Table S7. Strain-specific POGs annotated and classified of strain KIGAM252T using the KEGG database.

Number of Genes	K Number	Orthology Description	Pathway	Subcategory	Category
1	K00031	IDH1, IDH2, icd; isocitrate dehydrogenase [EC:1.1.1.42]	Citrate cycle (TCA cycle)	Carbohydrate metabolism	
			Carbon fixation pathways in prokaryotes	Energy metabolism	Metabolism
			Glutathione metabolism	Metabolism of other amino acids	
			Peroxisome	Transport and catabolism	Cellular Processes
1	K00100	bdhAB; butanol dehydrogenase [EC:1.1.1.-]	Butanoate metabolism	Carbohydrate metabolism	Metabolism
1	K00231	PPOX, hemY; protoporphyrinogen/coproporphyrinogen III oxidase [EC:1.3.3.4 1.3.3.15]	Porphyrin and chlorophyll metabolism	Metabolism of cofactors and vitamins	Metabolism
1	K00382	DLD, lpd, pdhD; dihydrolipoamide dehydrogenase [EC:1.8.1.4]	Glycolysis / Gluconeogenesis		
			Citrate cycle (TCA cycle)		
			Pyruvate metabolism	Carbohydrate metabolism	
			Glyoxylate and dicarboxylate metabolism		
			Propanoate metabolism		Metabolism
			Glycine, serine and threonine metabolism		
			Valine, leucine and isoleucine degradation	Amino acid metabolism	
			Lysine degradation		
			Tryptophan metabolism		
1	K00558	DNMT1, dcm; DNA (cytosine-5)-methyltransferase 1 [EC:2.1.1.37]	Cysteine and methionine metabolism	Amino acid metabolism	Metabolism
1	K00820	glmS, GFPT; glutamine---fructose-6-phosphate transaminase (isomerizing) [EC:2.6.1.16]	Amino sugar and nucleotide sugar metabolism	Carbohydrate metabolism	
			Alanine, aspartate and glutamate metabolism	Amino acid metabolism	Metabolism
			Insulin resistance	Endocrine and metabolic disease	
1	K00845	glk; glucokinase [EC:2.7.1.2]	Glycolysis / Gluconeogenesis		Metabolism
			Galactose metabolism	Carbohydrate metabolism	
			Starch and sucrose metabolism		

			Amino sugar and nucleotide sugar metabolism		
			Streptomycin biosynthesis	Biosynthesis of other secondary metabolites	
			Neomycin, kanamycin and gentamicin biosynthesis		
1	K00882	fruK; 1-phosphofructokinase [EC:2.7.1.56]	Fructose and mannose metabolism	Carbohydrate metabolism	Metabolism
			Phosphotransferase system (PTS)	Membrane transport	Environmental Information Processing
1	K00891	aroK, aroL; shikimate kinase [EC:2.7.1.71]	Phenylalanine, tyrosine and tryptophan biosynthesis	Amino acid metabolism	Metabolism
1	K01119	cpdB; 2',3'-cyclic-nucleotide 2'-phosphodiesterase / 3'-nucleotidase [EC:3.1.4.16 3.1.3.6]	Purine metabolism	Nucleotide metabolism	Metabolism
			Pyrimidine metabolism		
1	K01247	alkA; DNA-3-methyladenine glycosylase II [EC:3.2.2.21]	Base excision repair	Replication and repair	Genetic Information Processing
1	K01256	pepN; aminopeptidase N [EC:3.4.11.2]	Glutathione metabolism	Metabolism of other amino acids	Metabolism
1	K01443	nagA, AMDHD2; N-acetylglucosamine-6-phosphate deacetylase	Amino sugar and nucleotide sugar metabolism	Carbohydrate metabolism	Metabolism
1	K01448	amiABC; N-acetylmuramoyl-L-alanine amidase [EC:3.5.1.28]	Cationic antimicrobial peptide (CAMP) resistance	Drug resistance: antimicrobial	Human Diseases
1	K01793	GLCE; heparosan-N-sulfate-glucuronate 5-epimerase [EC:5.1.3.17]	Glycosaminoglycan biosynthesis - heparan sulfate / heparin	Glycan biosynthesis and metabolism	Metabolism
1	K01834	PGAM, gpmA; 2,3-bisphosphoglycerate-dependent phosphoglycerate mutase [EC:5.4.2.11]	Glycolysis / Gluconeogenesis	Carbohydrate metabolism	Metabolism
			Methane metabolism	Energy metabolism	
			Glycine, serine and threonine metabolism	Amino acid metabolism	
			Glucagon signaling pathway	Endocrine system	Organismal Systems
1	K01887	RARS, argS; arginyl-tRNA synthetase [EC:6.1.1.19]	Aminoacyl-tRNA biosynthesis	Translation	Genetic Information Processing
3	K01929	murF; UDP-N-acetylmuramoyl-tripeptide--D-alanyl-D-alanine ligase [EC:6.3.2.10]	Lysine biosynthesis	Amino acid metabolism	Metabolism
			Peptidoglycan biosynthesis	Glycan biosynthesis and metabolism	
			Vancomycin resistance	Drug resistance: antimicrobial	Human Diseases
1	K01955	carB, CPA2; carbamoyl-phosphate synthase large subunit [EC:6.3.5.5]	Pyrimidine metabolism	Nucleotide metabolism	Metabolism

			Alanine, aspartate and glutamate metabolism	Amino acid metabolism	
1	K02250	comK; competence protein ComK	Quorum sensing	Cellular community - prokaryotes	Cellular Processes
1	K02398	flgM; negative regulator of flagellin synthesis FlgM	Two-component system	Signal transduction	Environmental Information Processing
			Biofilm formation - Pseudomonas aeruginosa	Cellular community - prokaryotes	Cellular Processes
			Biofilm formation - Escherichia coli		
			Flagellar assembly	Cell motility	
1	K02414	fliK; flagellar hook-length control protein FliK	Flagellar assembly	Cell motility	Cellular Processes
1	K02488	pleD; two-component system, cell cycle response regulator [EC:2.7.7.65]	Two-component system	Signal transduction	Environmental Information Processing
			Cell cycle - Caulobacter	Cell growth and death	Cellular Processes
1	K02491	kinA; two-component system, sporulation sensor kinase A [EC:2.7.13.3]	Two-component system	Signal transduction	Environmental Information Processing
1	K03070	secA; preprotein translocase subunit SecA [EC:7.4.2.8]	Protein export	Folding, sorting and degradation	Genetic Information Processing
			Bacterial secretion system	Membrane transport	Environmental Information Processing
			Quorum sensing	Cellular community - prokaryotes	Cellular Processes
1	K03148	thiF; sulfur carrier protein ThiS adenylyltransferase [EC:2.7.7.73]	Thiamine metabolism	Metabolism of cofactors and vitamins	Metabolism
			Sulfur relay system	Folding, sorting and degradation	Genetic Information Processing
1	K03657	uvrD, pcrA; DNA helicase II / ATP-dependent DNA helicase PcrA [EC:3.6.4.12]	Nucleotide excision repair	Replication and repair	Genetic Information Processing
			Mismatch repair		
1	K04565	SOD1; superoxide dismutase, Cu-Zn family [EC:1.15.1.1]	Peroxisome	Transport and catabolism	Cellular Processes
			Longevity regulating pathway - multiple species	Aging	Organismal Systems
1	K05845	opuC; osmoprotectant transport system substrate-binding protein	ABC transporters	Membrane transport	Environmental Information Processing
2	K05846	opuBD; osmoprotectant transport system permease protein	ABC transporters	Membrane transport	Environmental Information Processing
1	K05847	opuA; osmoprotectant transport system ATP-binding protein [EC:7.6.2.9]	ABC transporters	Membrane transport	Environmental Information Processing

1	K05901	BLVRB; biliverdin reductase / flavin reductase [EC:1.3.1.24 1.5.1.30]	Riboflavin metabolism	Metabolism of cofactors and vitamins	Metabolism
			Porphyrin and chlorophyll metabolism		
1	K06236	COL1A; collagen type I alpha	PI3K-Akt signaling pathway	Signal transduction	Environmental Information Processing
			ECM-receptor interaction	Signaling molecules and interaction	
			Focal adhesion	Cellular community - eukaryotes	Cellular Processes
			Platelet activation	Immune system	Organismal Systems
			Relaxin signaling pathway	Endocrine system	
1	K07697	kinB; two-component system, sporulation sensor kinase B [EC:2.7.13.3]	Two-component system	Signal transduction	Environmental Information Processing
1	K07816	E2.7.6.5X; putative GTP pyrophosphokinase [EC:2.7.6.5]	Purine metabolism	Nucleotide metabolism	Metabolism
1	K08093	3-hexulose-6-phosphate synthase [EC:4.1.2.43]	Pentose phosphate pathway	Carbohydrate metabolism	Metabolism
			Methane metabolism	Energy metabolism	
1	K08302	gatY-kbaY; tagatose 1,6-diphosphate aldolase GatY/KbaY [EC:4.1.2.40]	Galactose metabolism	Carbohydrate metabolism	Metabolism
1	K08693	yfkN; 2',3'-cyclic-nucleotide 2'-phosphodiesterase / 3'-nucleotidase / 5'-nucleotidase [EC:3.1.4.16 3.1.3.6 3.1.3.5]	Purine metabolism	Nucleotide metabolism	Metabolism
			Pyrimidine metabolism		
			Nicotinate and nicotinamide metabolism	Metabolism of cofactors and vitamins	
1	K10439	rbsB; ribose transport system substrate-binding protein	ABC transporters	Membrane transport	Environmental Information Processing
			Bacterial chemotaxis	Cell motility	Cellular Processes
1	K10441	rbsA; ribose transport system ATP-binding protein [EC:7.5.2.7]	ABC transporters	Membrane transport	Environmental Information Processing
1	K11294	NCL, NSR1; nucleolin	Pathogenic Escherichia coli infection	Infectious disease: bacterial	Human Diseases
1	K14755	CRNS1, ATPGD1; carnosine synthase [EC:6.3.2.11]	Arginine and proline metabolism	Amino acid metabolism	Metabolism
			Histidine metabolism		
			beta-Alanine metabolism	Metabolism of other amino acids	
1	K15634	gpmB; 2,3-bisphosphoglycerate-dependent phosphoglycerate mutase [EC:5.4.2.11]	Glycolysis / Gluconeogenesis	Carbohydrate metabolism	Metabolism
			Methane metabolism	Energy metabolism	

			Glycine, serine and threonine metabolism	Amino acid metabolism	
1	K16012	cydC; ATP-binding cassette, subfamily C, bacterial CydC	ABC transporters	Membrane transport	Environmental Information Processing
1	K16013	cydD; ATP-binding cassette, subfamily C, bacterial CydD	ABC transporters	Membrane transport	Environmental Information Processing
1	K16919	ytrC_D; acetoin utilization transport system permease protein	ABC transporters	Membrane transport	Environmental Information Processing
1	K17318	K17318, lplA; putative aldouronate transport system substrate-binding protein	ABC transporters	Membrane transport	Environmental Information Processing
1	K00375	K00375; GntR family transcriptional regulator / MocR family aminotransferase	-	-	-
1	K00517	CYP81F; indol-3-yl-methylglucosinolate hydroxylase [EC:1.14.-.-]	-	-	-
2	K00661	maa; maltose O-acetyltransferase [EC:2.3.1.79]	-	-	-
1	K01153	hsdR; type I restriction enzyme, R subunit [EC:3.1.21.3]	-	-	-
1	K01154	hsdS; type I restriction enzyme, S subunit [EC:3.1.21.3]	-	-	-
2	K01174	nuc; micrococcal nuclease [EC:3.1.31.1]	-	-	-
1	K01308	yqgT; g-D-glutamyl-meso-diaminopimelate peptidase [EC:3.4.19.11]	-	-	-
1	K01342	aprE; subtilisin [EC:3.4.21.62]	-	-	-
1	K01421	yhgE; putative membrane protein	-	-	-
3	K03088	rpoE; RNA polymerase sigma-70 factor, ECF subfamily	-	-	-
1	K03293	TC.AAT; amino acid transporter, AAT family	-	-	-
1	K03427	hsdM; type I restriction enzyme M protein [EC:2.1.1.72]	-	-	-
1	K03574	mutT, NUDT15, MTH2; 8-oxo-dGTP diphosphatase [EC:3.6.1.55]	-	-	-
1	K03593	mrp, NUBPL; ATP-binding protein involved in chromosome partitioning	-	-	-
1	K03603	fadR; GntR family transcriptional regulator, negative regulator for fad regulon and positive regulator of fabA	-	-	-
1	K03631	recN; DNA repair protein RecN (Recombination protein N)	-	-	-
1	K03710	K03710; GntR family transcriptional regulator	-	-	-
1	K03761	kgtP; MFS transporter, MHS family, alpha-ketoglutarate permease	-	-	-
1	K03893	arsB; arsenical pump membrane protein	-	-	-

1	K04750	phnB; PhnB protein	-	-	-
1	K04758	feoA; ferrous iron transport protein A	-	-	-
3	K04759	feoB; ferrous iron transport protein B	-	-	-
1	K04763	xerD; integrase/recombinase XerD	-	-	-
1	K05593	aadK; aminoglycoside 6-adenylyltransferase [EC:2.7.7.-]	-	-	-
1	K06023	hprK, ptsK; HPr kinase/phosphorylase [EC:2.7.11.- 2.7.4.-]	-	-	-
2	K06147	ABCB-BAC; ATP-binding cassette, subfamily B, bacterial	-	-	-
1	K06318	bofC; forespore regulator of the sigma-K checkpoint	-	-	-
1	K06325	cotB; spore coat protein B	-	-	-
1	K06330	cotH; spore coat protein H	-	-	-
1	K06388	spoIISA; stage II sporulation protein SA	-	-	-
1	K07038	K07038; inner membrane protein	-	-	-
1	K07039	K07039; uncharacterized protein	-	-	-
1	K07120	K07120; uncharacterized protein	-	-	-
1	K07124	K07124; uncharacterized protein	-	-	-
1	K07152	SCO1; protein SCO1	-	-	-
1	K07243	FTR, FTH1, efeU; high-affinity iron transporter	-	-	-
1	K07453	K07453; putative restriction endonuclease	-	-	-
2	K07507	mgtC; putative Mg2+ transporter-C (MgtC) family protein	-	-	-
1	K08071	NQO2; ribosyldihydronicotinamide dehydrogenase (quinone) [EC:1.10.5.1]	-	-	-
1	K08884	K08884; serine/threonine protein kinase, bacterial [EC:2.7.11.1]	-	-	-
1	K09681	gltC; LysR family transcriptional regulator, transcription activator of glutamate synthase operon	-	-	-
1	K09702	K09702; uncharacterized protein	-	-	-
1	K09962	K9962: uncharacterized protein	-	-	-
1	K11162	RDH14; retinol dehydrogenase 14 [EC:1.1.1.-]	-	-	-
1	K13274	wprA; cell wall-associated protease [EC:3.4.21.-]	-	-	-
1	K13530	adaA; AraC family transcriptional regulator, regulatory protein of adaptative response / methylphosphotriester-DNA alkyltransferase methyltransferase [EC:2.1.1.-]	-	-	-
1	K16264	czcD, zitB; cobalt-zinc-cadmium efflux system protein	-	-	-
1	K17803	OMS1; methyltransferase OMS1, mitochondrial [EC:2.1.1.-]	-	-	-

1	K06438	yqfD; similar to stage IV sporulation protein	-	-	-
1	K19428	epsL; sugar transferase EpsL	-	-	-

Table S8. Carotenoid identification based on chromatographic and spectral properties of carotenoids detected in extracts of strain KIGAM252^T.

**Peak**	**HPLC**	**Spectral Properties**	**Carotenoid Prediction**
	R_t (min)	λ (nm)	
Trace at 450 nm (Fig. 6A)			
1	36.54	428, 448, 476	
2	37.24	428, 448, 476	*cis*-1-glycosyl-apo-8'-lycopene
3	36.70	428, 448, 476	
4	38.38	430, 454, 484	1-glycosyl-apo-8'-lycopene
5	38.68	430, 454, 484	
Trace at 286 nm (Fig. 6B)			
6	23.62	274, 286, 298	
7	24.33	274, 286, 298	Apo-8'-phytoene
8	26.51	274, 286, 298	

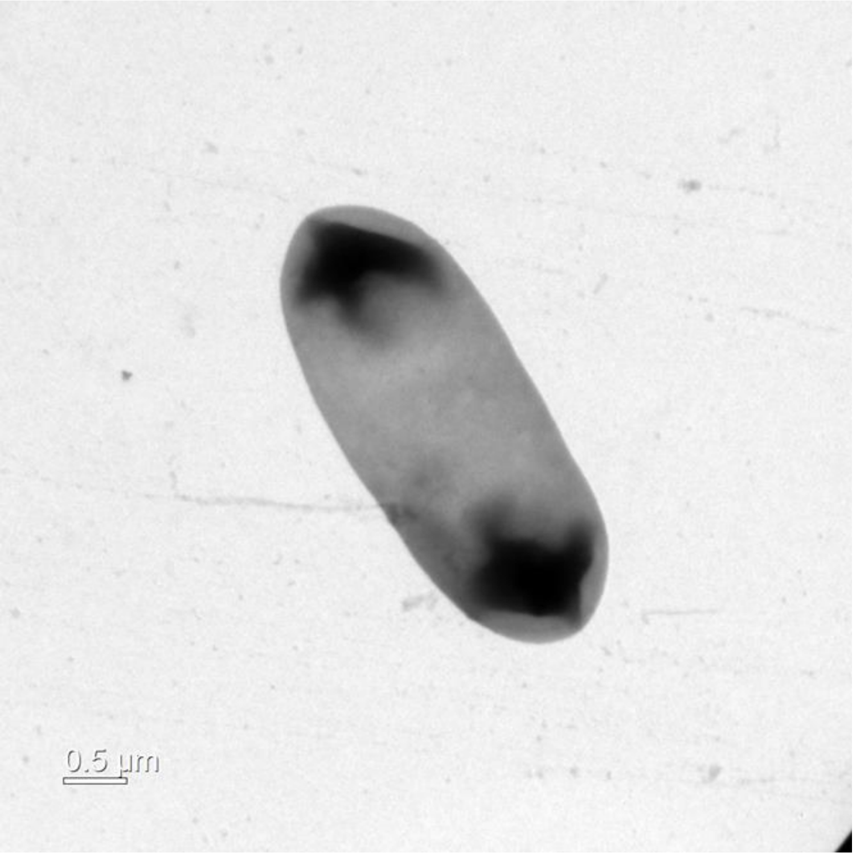

Figure S1. Transmission electron micrograph of negatively stained strain KIGAM252^T. Strain KIGAM252^T was cultivated at 30 °C for two days in TSA. Bar, 0.5 µm.

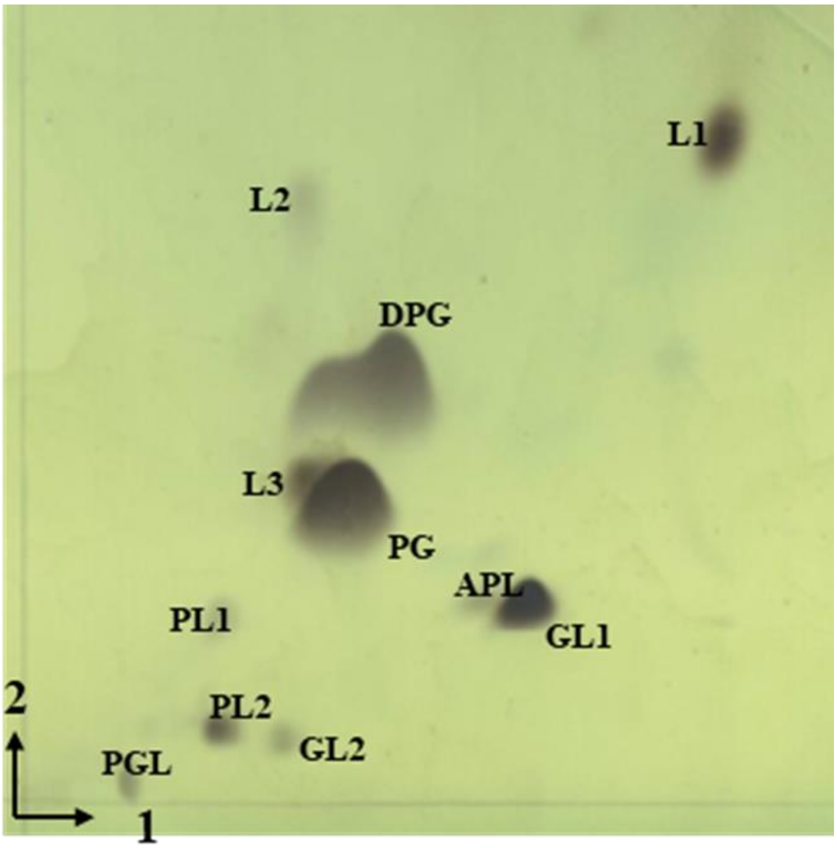

Figure S2. Thin-layer chromatograms of the polar lipids of strain KIGAM252^{T}. The 1st-D developing agent, chloroform:methanol:water (65:25:4, v/v/v) and the 2nd-D agent, chloroform:acetic acid:methanol:water (80:15:12:4, v/v/v/v). The major polar lipids are diphosphatidylglycerol (DPG) and phosphatidylglycerol (PG). PGL, phosphatidylglycolipid; APL, aminophospholipid; PL, unidentified phospholipid; GL, unidentified glycolipid; L, unidentified lipid.

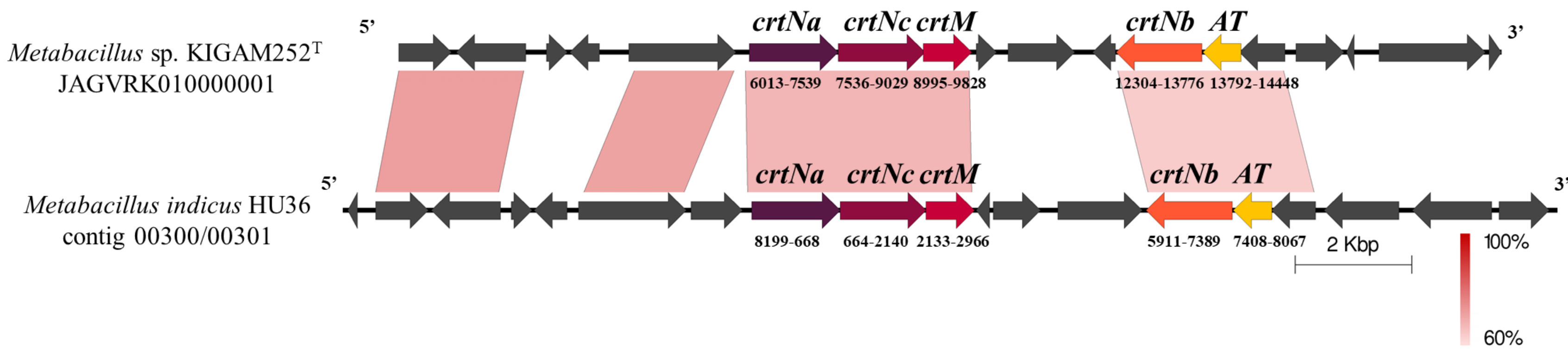

Figure S3. Carotenoid biosynthetic gene cluster comparison between strain KIGAM252^T and *M. indicus* HU36 based on the nucleotide sequence similarities. The shaded pink areas between linear structures indicate homologous regions. The gene: *crtM*, 4,4' -diapophytoene synthase; *crtNa*, 4,4' -diapophytoene desaturase; *crtNb*, 4,4' -diapophytoene-ketolase; *crtNc*, 4,4' -diapophytoene aldehyde oxidase; *AT*, acyltransferase. The genes indicated by gray arrows represent those that have no relationship with carotenoid production.

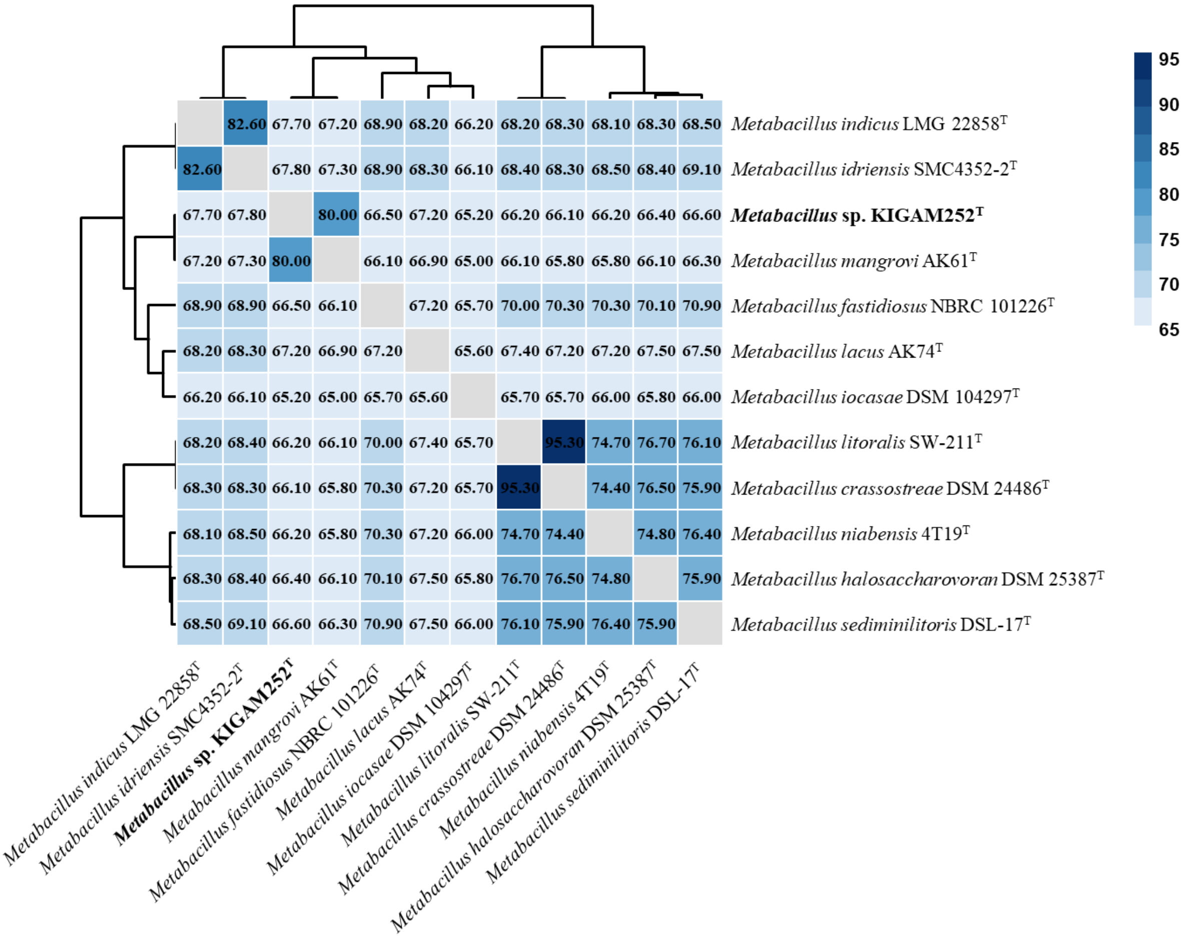

Figure S4. Heatmap based on AAI values calculated for strainKIGAM252^T and other species of the genus *Metabacillus*; *M. mangrovi* AK61^T, *M. indicus* LMG 22858^T, *M. idriensis* SMC4352-2^T, *M. lacus* AK74^T, *M. sediminilitoris* DSL-17^T, *M. fastidiosus* NBRC 101226^T, *M. halosaccharovorans* DSM 25387^T, *M. niabensis* 4T19^T, *M. crassostreae* DSM 24486^T, *M. litoralis* SW-2211^T, and *M. iocasae* DSM 104297^T. High AAI value is indicated in navy, whereas lower value is indicated in sky blue.

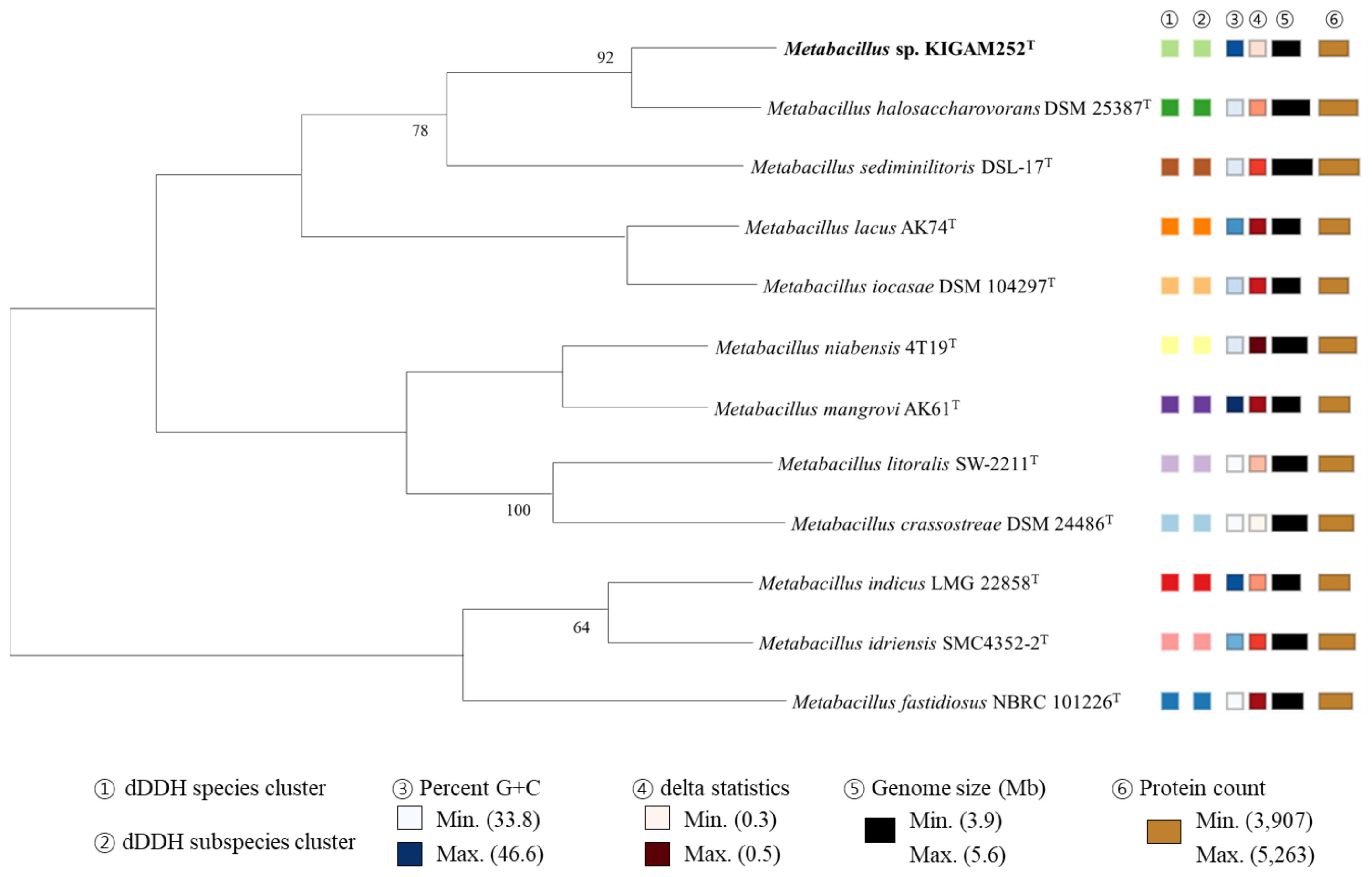

Figure S5. Phylogenomic tree based on TYGS results showing the relationship between strain KIGAM252^{T} with related type strains in genus *Metabacillus*. The whole-genome sequence-based tree was inferred with FastME 2.1.6.1 (Lefort et al., 2015) from GBDP distances. calculated from genome sequences. The branch lengths are scaled in terms of GBDP distance formula d5. The numbers above branches are GBDP pseudo-

bootstrap support values > 60 % from 100 replications, with average branch support of 98.7 %. Percent of genomic G+C content ranges between 33.8 and 46.6 %; genome size ranges from 3.9 to 5.6 Mb; The number of proteins coded by each genome ranges between 3,907 and 5,263.

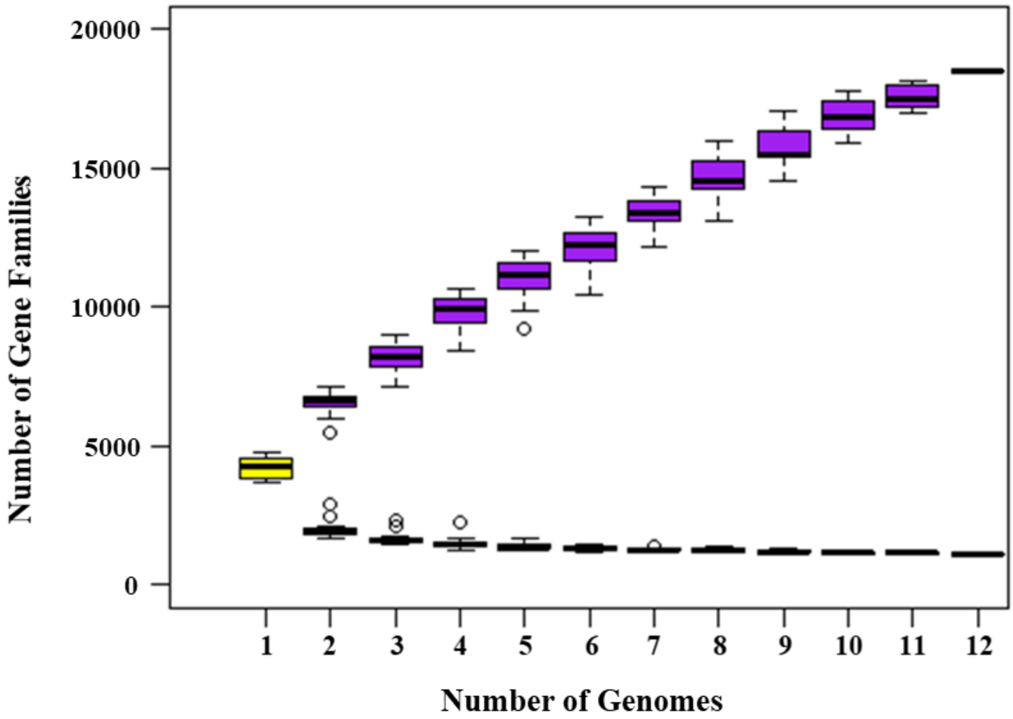

Figure S6. Pan-genome and clustering analysis of strain KIGAM252[T] and other species of the genus *Metabacillus*. The boxplots of the pan (purple color) and core genomes (yellow color) are progressively increasing or decreasing by the number of genomes.

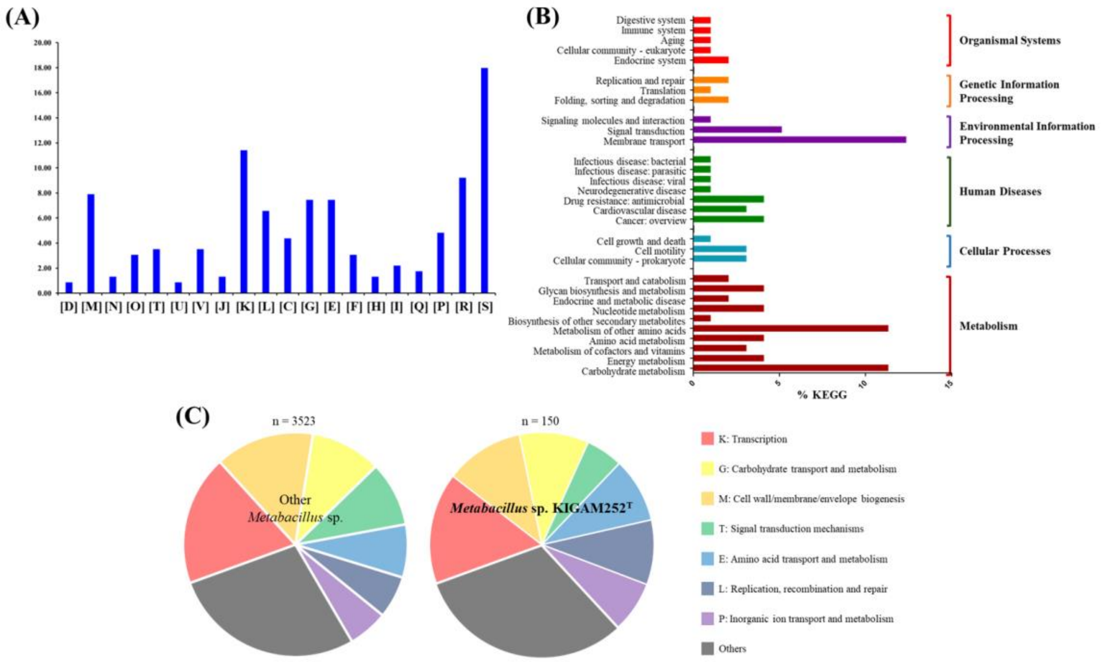

Figure S7. COG and KEGG categorization of unique genes of strain KIGAM252^T. The strain-specific functional unique POG annotation of strain KIGAM252^T using COG database (**A**) and The strain-specific functional unique POG annotation of strain KIGAM252^T using the KEGG database (**B**). Fraction of unique genes associated with different COG categories for strain KIGAM252^T and related strains of the genus *Metabacillus*; *M. mangrovi* AK61^T, *M. indicus* LMG 22858^T, *M. idriensis* SMC4352-2^T, *M. lacus* AK74^T, *M. sediminilitoris* DSL-17^T, *M. fastidiosus* NBRC 101226^T, *M. halosaccharovorans* DSM 25387^T, *M. niabensis* 4T19^T, *M. crassostreae* DSM 24486^T, *M. litoralis* SW-2211^T, and *M. iocasae* DSM 104297^T (**C**). One-letter abbreviations for the COG categories: J, translation, ribosomal structure and biogenesis; K, transcription; L, replication, recombination, and repair; B, chromatin structure and dynamics; D, cell cycle control, cell division, chromosome partitioning; V, defense mechanisms; T, signal transduction mechanisms; M, cell wall/membrane/envelope biogenesis; N, cell motility; U, intracellular trafficking, secretion, and vesicular transport; O, post-translational modification, protein turnover, and chaperones; C, energy production and conversion; G, carbohydrate transport and metabolism; E, amino acid transport and metabolism; F, nucleotide transport and

metabolism; H, coenzyme transport and metabolism; I, lipid transport and metabolism; P, inorganic ion transport and metabolism; Q, secondary metabolites biosynthesis, transport; R, general function prediction only, and catabolism; S, function unknown.

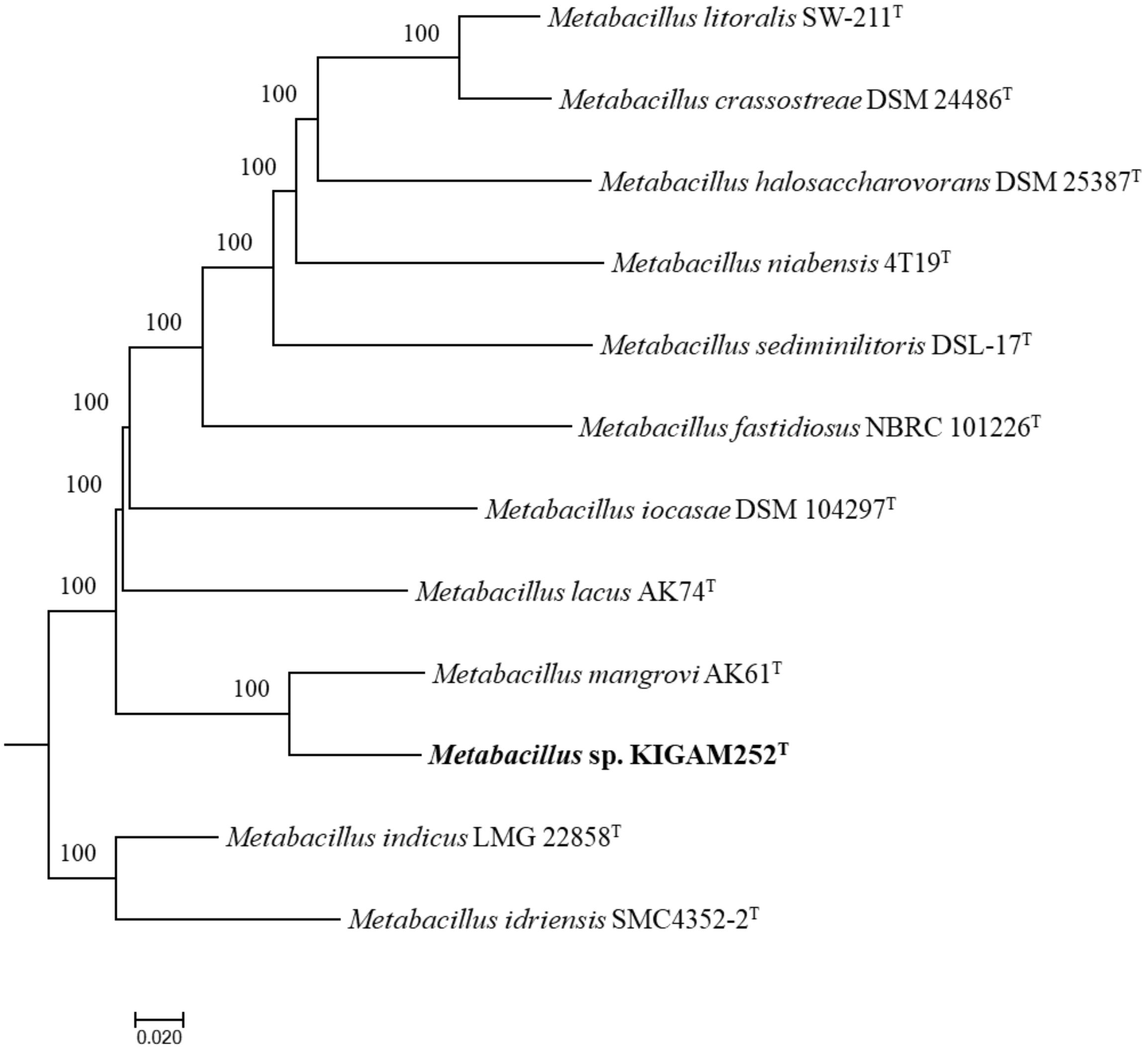

Figure S8. Phylogenomic tree using concatenated 347 core genes of strain KIGAM252^T and other species of the genus *Metabacillus*. The tree was constructed using the NJ method with 1,000 bootstrap replications.
